# Inside the Atacama Desert: uncovering the living microbiome of an extreme environment

**DOI:** 10.1128/aem.01443-24

**Published:** 2024-11-14

**Authors:** Alexander Bartholomäus, Steffi Genderjahn, Kai Mangelsdorf, Beate Schneider, Pedro Zamorano, Samuel P. Kounaves, Dirk Schulze-Makuch, Dirk Wagner

**Affiliations:** 1 GFZ German Research Centre for Geosciences, Section Geomicrobiology, Potsdam, Germany; 2 GFZ German Research Centre for Geosciences, Section Organic Geochemistry, Potsdam, Germany; 3 Center of Astronomy and Astrophysics, Technical University Berlin, Berlin, Germany; 4 Laboratorio de Microorganismos Extremófilos, University of Antofagasta, Antofagasta, Chile; 5 Department of Chemistry, Tufts University, Medford, Massachusetts, USA; 6 Department of Earth Science & Engineering, Imperial College London, London, United Kingdom; 7 Department of Experimental Limnology, Leibniz-Institute of Freshwater Ecology and Inland Fisheries (IGB), Stechlin, Germany; 8 University of Potsdam, Institute of Geosciences, Potsdam, Germany; Georgia Institute of Technology, Atlanta, Georgia, USA

**Keywords:** microbial diversity, DNA extraction, extracellular DNA, intracellular DNA, Atacama Desert, specialist, microbiome

## Abstract

The Atacama Desert in Chile is one of the driest and most inhospitable places on Earth. To analyze the diversity and distribution of microbial communities in such an environment, one of the most important and challenging steps is DNA extraction. Using commercial environmental DNA extraction protocols, a mixture of living, dormant, and dead cells of microorganisms is extracted, but separation of the different DNA pools is almost impossible. To overcome this problem, we applied a novel method on soils across a west–east moisture transect in the Atacama Desert to distinguish between extracellular DNA (eDNA) and intracellular DNA (iDNA) at the cell extraction level. Here, we show that a large number of living and potentially active microorganisms, such as *Acidimicrobiia*, *Geodermatophilaceae*, *Frankiales*, and *Burkholderiaceae,* occur in the hyperarid areas. We observed viable microorganisms involved as pioneers in initial soil formation processes, such as carbon and nitrogen fixation, as well as mineral-weathering processes. In response to various environmental stressors, microbes coexist as generalists or specialists in the desert soil environment. Our results show that specialists compete in a limited range of niches, while generalists tolerate a wider range of environmental conditions. Use of the DNA separation approach can provide new insights into different roles within viable microbial communities, especially in low-biomass environments where RNA-based analyses often fail.

**IMPORTANCE:**

The novel e- and iDNA separation technique offers insights into the living community at the cell extraction level in the hyperarid Atacama Desert. This approach provides a new framework for analyzing the composition and structure of the potentially active part of the microbial communities as well as their specialization, ecological network and community assembly process. Our findings underscore the significance of utilizing alternative genomic techniques in low-biomass environments where traditional DNA- and RNA-based analyses may not be feasible. The results demonstrate the viability of the proposed study framework and show that specialized microorganisms are important in initial soil formation processes, including microbial-driven mineral weathering, as well as the fixation of carbon and nitrogen.

## INTRODUCTION

The Atacama Desert in northern Chile is one of the driest and oldest deserts on Earth, with a stable climate and a surface that has been barely disturbed by erosion over the past 150 million years (1–3). The extreme aridity is a result of the strong Pacific anticyclone and the cold north-flowing Humboldt Current. This combination leads to a constant temperature inversion, offshore winds, and a rain shadow from the Andes to the east (4–6). The most hyperarid zone of the Atacama Desert, located ~60 km inland, has a mean annual precipitation of about 2 mm year^−1^, and rare rainfall events typically occurring only once per decade during El Niño years (1, 4, 7).

This extreme aridity is one reason why it is considered the most hostile environment with regard to the dry limit for life on Earth (8). The main source of water in this region is coastal fog, though most of it is blocked by the western mountain range, the Cordillera de la Costa, and thus water availability decreases with increasing distance from the coast (9). The rare fog events that do occur provide little moisture (10). As a result the sparsely vegetated ground surfaces are located only in the coastal region or where groundwater occurs *via* local springs (11).

The development of this extremely dry climate has led to the accumulation of chloride, nitrate, sulfate, and perchlorate, bearing minerals, from tropospheric and eolian depositional processes, and has resulted in massive salt-indurated soil horizons that are overlain by unconsolidated sulfate-rich topsoil horizons (12). In addition, the environmental conditions in the Atacama Desert are characterized by wide daily temperature fluctuations (0°C–32°C), the world’s highest levels of surface UV radiation (3.5–5 kWh m^−2^), and extremely low organic carbon content of the soils of <0.1% (13, 14).

Nevertheless, the Atacama Desert is colonized by abundant and diverse microbial communities. Previous studies found culturable bacteria in the range from almost 0 to 10^6^ colony forming units (CFU) g^−1^ soil, reflecting a large spatial heterogeneity (8, 15). Modern DNA-based analyses have shown a wide range of gene copy numbers for bacteria and archaea, from 10^4^ to 10^8^ and 10^4^ to 10^6^ g^−1^ soil, respectively (14, 16). A characterization of the individual communities by sequencing 16S rRNA gene clone libraries of top soil horizons performed by Connon *et al.* (15) revealed Actinobacteria as the dominant phylum along with low abundances of *Saccharibacteria* (formerly known as candidate phylum TM7), Firmicutes, and Proteobacteria. A study by Neilson *et al.* (17) focused on bacterial communities in soil (15–25 cm depth) in the transition zone from arid to hyperarid soils along a west–east elevational transect. Crits-Christoph *et al.* (18) characterized soil microbial communities (0–10 cm depth) along a north–south moisture gradient. Both of these studies confirmed the high abundance of Actinobacteria, typical of desert soils (19–21), but differed with regard to composition and abundances of less frequent phyla and classes essentially comprising Proteobacteria, Acidobacteria, Bacteroidetes, Chloroflexi, Firmicutes, Saccharibacteria, Gemmatimonadetes, Thermomicrobia, Tenericutes, and Nitrospira. The results showed that the overall diversity of microbial communities in these arid to hyperarid soils decreases with decreasing humidity.

Although in recent years great progress has been made in microbial community analyses in extreme environments, conventional DNA- or RNA-based methods often fail due to the low yield of extractable genomic material in desert soils (8, 22, 23). The matter of fact that microbial cells can only be intermittently active in the desert’s environment (14) leads to an overall low biomass and extremely low RNA levels. To the best of our knowledge, no metatranscriptome analysis from the Atacama Desert has yet been published that could provide insights into the functional diversity in this extreme habitat. Nevertheless, it is possible to extract total DNA from desert soils using common methods, which obviously include DNA from living, dormant, and dead cells. Based on the total DNA extract, it is therefore not possible to distinguish whether the DNA originates from living microorganisms (intracellular DNA, iDNA) or represents DNA that is preserved “naked” in the sediment (extracellular DNA, eDNA) and thus represents dead microorganisms. Differentiation of these two DNA pools may provide insights into the living and potentially active microorganisms of the desert microbiome independently of transcriptome analyses. Furthermore, distinguishing between iDNA and eDNA is critical for improving the resolution of DNA data in low biomass environments and to avoid misinterpretation of the diversity of the actual living microbial community (24, 25). Therefore, the separation into these two DNA pools can be a valuable attribute when studying the microbial diversity of original habitats, particularly in low-biomass ecosystems.

To study ecological key organisms, specialist and generalist analyses have been performed for different environments (26, 27). Specialists are organisms that might be highly adapted to specific environmental condition that might be especially important in extreme environments. Generalists are often considered to be very flexible and adaptable and able to cope with different conditions (28). Together, with the iDNA extraction methods used here, the detection of specialist could give a more reliable picture of organisms that are currently living.

In the present study, we investigated the microbial community in detail along a moisture transect and two hyperarid reference sites in the Atacama Desert. We combined molecular biological data obtained with the DNA separation approach with environmental parameters to determine important community-shaping parameters, and thus better understand hyperarid ecosystems and their associated diverse living microbial communities. In addition, we characterized generalists and specialists by focusing on their key roles in adapting to environmental variation.

## RESULTS

### Bacterial abundance along the studied moisture transects

**Fig 1:**
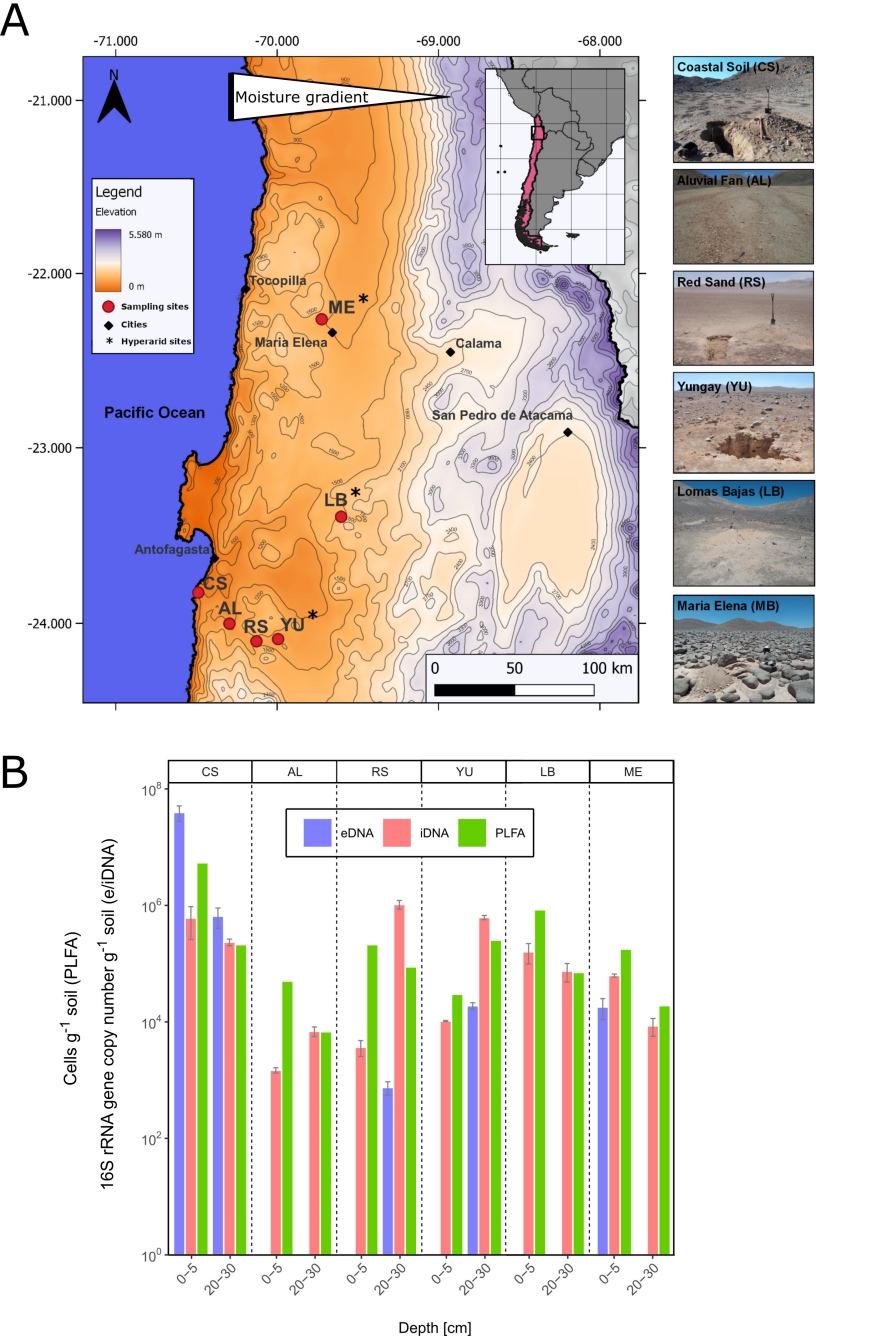
Location of the study sites and bacterial abundances. (**A**) Location of the study sites along the Atacama Desert. Moisture gradient including Coastal Sand (CS), Alluvial Fan (AL), Red Sands (RS), Yungay (YU), and two hyperarid reference sites, Maria Elena (ME) and Lomas Bayas (LB). (**B**) Bacterial abundance based on 16S rRNA gene copy numbers of the e- and iDNA pools (means ± SE, *n* = 3–4, see Table S1), and phospholipid fatty acids (PLFAs) in the different investigation sites along the Atacama transect. Missing gene copy numbers for the eDNA pool indicate less than three replicates for the respective study site.

The bacterial gene copy numbers for both DNA pools and the microbial biomass calculated from phospholipid fatty acids (PLFA) have been determined along the fog related W-E moisture transects, namely Coastal Sand (CS), Aluvial Fan (AL), Red Sand (RS), and Yungay (YU), and two additional hyperarid reference sites, Maria Elena (ME) and Lomas Bayas (LB) (Fig. 1A). The microbial abundance was determined at two depth intervals (0–5 and 20–30 cm) for all sites.

The numbers for eDNA varied between 7.5(±1.9) × 10^2^ 16S rRNA gene copies g^−1^ soil in RS and 3.9(±1.2) × 10^7^ gene copies g^−1^ soil (Fig. 1B) in CS. For some eDNA extracts (*e.g.*, AL, LB), it was not possible to obtain qPCR results (Table S1). The gene copy number of the iDNA varied less compared with the eDNA, between 6.9(±1.3) × 10^3^ gene copies g^−1^ soil in AL and 1.0(±0.18) × 10^6^ gene copies g^−1^ soil in RS. Based on the PLFA concentration, complimentary cell numbers were calculated, which varied between 6.8 × 10^3^ cells g^−1^ soil at the 20–30 cm soil depth in AL and 5.4 × 10^6^ cells g^−1^ soil in the soil surface of CS. The PLFA values were in most cases in good agreement with the gene copy numbers in the iDNA pool. No general trend in the bacterial abundances along the moisture transect was seen, except that the surface bacterial community at the coastal site CS is more abundant than in the desert location. For the subsurface community such a trend is not obvious.

### General composition of microbial communities

The microbial community composition was analyzed by high-throughput sequencing for all study sites at the two soil depth intervals (0–5 and 20–30 cm), and with a differentiation between eDNA and iDNA (each in triplicates). A total of 72 samples resulted in 4,239,159 sequencing reads. After demultiplexing, filtering, denoising, merging the forward and reverse reads, and chimeric removal, 2,835,790 reads remained in the final data set, which corresponds to 66.9% of all sequences (Table S2). These sequences were distributed to 49% in the eDNA pool and 51% in the iDNA pool. The number of reads per sample ranged from 14,515 to 106,973 with a mean value of 38,846. In total, 96.1% of all amplicon sequence variants (ASVs) were assigned to Bacteria, 3.8% to Archaea, and 0.1% others. After taxonomic classification, 6,205 putative taxa (5,965 for Bacteria, 235 for Archaea) were obtained.

The Shannon, Chao, and Simpson indices, were used to estimate and compare the alpha diversity of the different samples along the moisture transect (Fig. S1). The Shannon index ranged from 6.5 to 2.9, with the highest alpha diversity found in the surface sample (0–5 cm) of CS.

The microbial communities at the two sampling depths (0–5 and 20–30 cm) along the transect were characterized by six *dominant phyla* (group defined as relative abundance ≥10%; total coverage 70.2%–93.5%; Fig. S2; Table S3): Actinobacteria, Proteobacteria, Chloroflexi, Cyanobacteria, Firmicutes, and Euryarchaeota. While Actinobacteria (10.1%–70.9%) and Proteobacteria (11.0%–81.9%) occur virtually at all locations and depth intervals, the phototrophic groups Chloroflexi (10.4%–30.3%) and Cyanobacteria (11.1%–33.5%) only appear as *dominant phyla* in the surface samples. The distribution of Firmicutes (10.8%–47.4%) showed no clear trend. ASVs of the archaeal phylum Euryarchaeota (34.1%–59.9%) were only detected at CS at the 20–30 cm soil depth, but with highest abundance in both DNA pools. Actinobacteria and Proteobacteria were found in all samples in both DNA pools. In contrast, Chloroflexi dominated in the iDNA pool at a depth of 0–5 cm at all study sites except AL, while all other *dominant phyla* showed no clear trend in the distribution between the e- and iDNA pools.

In contrast to the *dominant phyla* (abundance ≥10%), another 15 *less dominant phyla* with an abundance between 1% and 9.9% (total coverage 3.3%–27.1%; Fig. S2; Table S3) were present. It should be noted that the six *dominant phyla* defined above have a lower relative abundance (<10%) in some other study sites/samples and thus also fall into the group of *less dominant phyla* at these sites. Of the 15 *less dominant phyla*, Firmicutes (2.0%–8.5%), Chloroflexi (1.1%–6.6%), Cyanobacteria (1.1%–8.2%), Gemmatimonadetes (1.1%–8.7%), and Bacteriodetes (1.0%–4.4%) were the most common, which occurred in more than 10 samples out of 24 for the upper two depth intervals. Firmicutes were found in almost all samples and thus have the largest distribution overall, followed by Cyanobacteria. The *less dominant phyla* are Cyanobacteria and Chloroflexi, especially at a depth of 20–30 cm in both DNA pools and in all study sites with the exception of CS, while they were dominant in the surface layer as described above. Gemmatimonadetes (1.1%–8.7%) occurred predominantly at a soil depth of 20–30 cm, especially in the iDNA pool of all sites with the exception of AL. Bacteroidetes (1.0%–4.4%) were found in the surface samples of CS and AL in both DNA pools. Moreover, Bacteroidetes occurred in the eDNA pool at a 20–30 cm soil depth of all sites except RS. In addition to the Euryarchaeota occurring as a *dominant phylum*, with the Thaumarchaeota (1.0%–1.2%), another archaeal group was detected as a *less dominant phyla*, which appeared only in the iDNA pool of the surface sample from CS. All other phyla (Acidobacteria, Actinobacteria, Deinococcus-Termus, Euryarchaeota, Patescibacteria, Planctomycetes, Proteobacteria, and Spirochaetes) were detected in only a few samples, without a clear trend regarding study sites or DNA pools.

**Fig 2:**
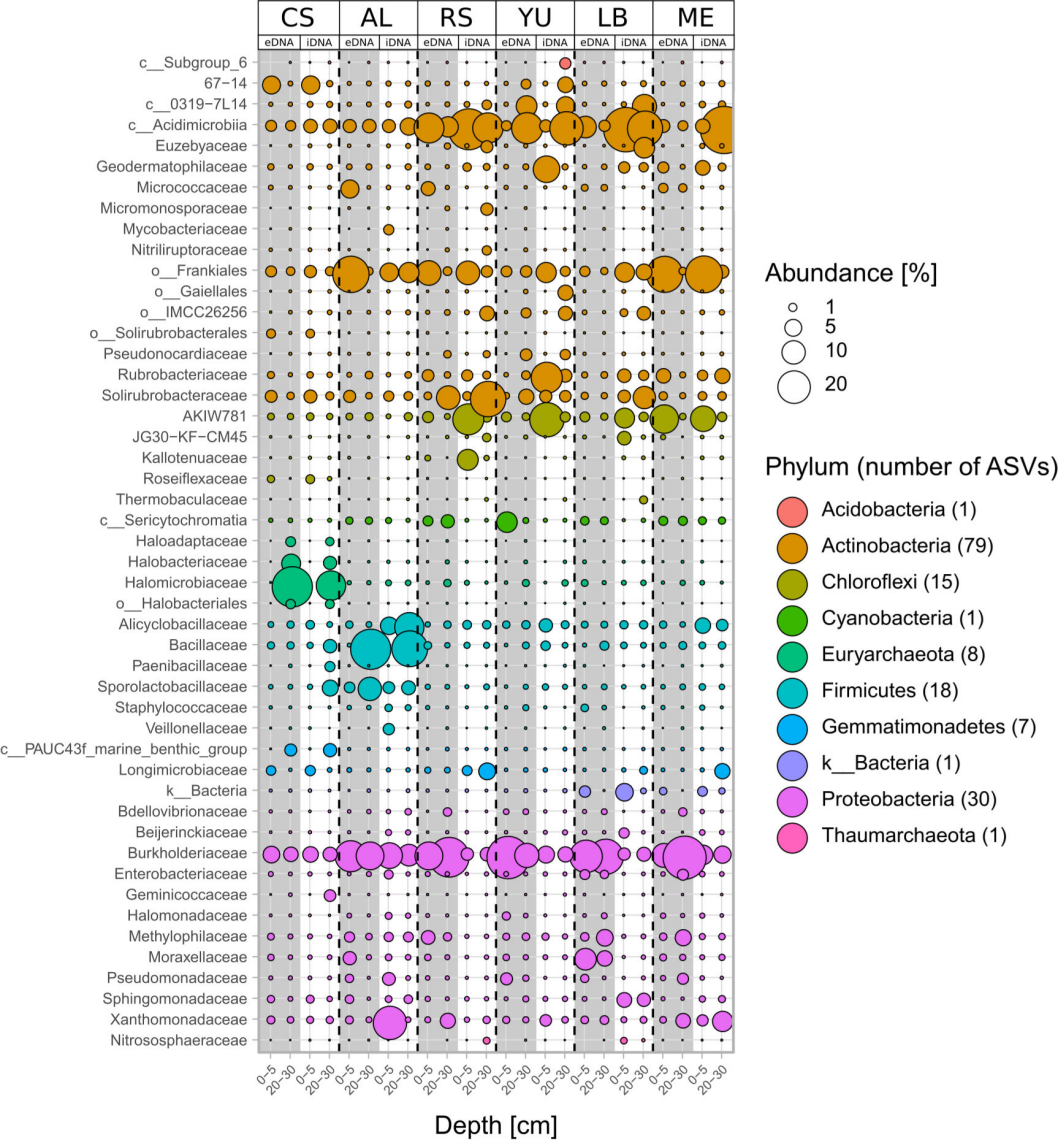
Relative abundances of the top 161 ASVs (>0.1% mean abundance) summarized by family level for the e- and iDNA pools along the moisture gradient: Coastal Sand (CS), Aluvial Fan (AL), Red Sands (RS), Yungay (YU), and additional hyperarid study sites Lomas Bayas (LB) and Maria Elena (ME). Bubbles represent the mean value of relative abundances from three technical replicates. ASVs are sorted by phylum and characterized by their family name; otherwise, the next highest classification level was chosen (o = order, c = class). k_Bacteria represents an unknown environmental clone related to Firmicutes (86% similarity) and Chloroflexi (87% similarity).

The 161 most abundant ASVs (> 0.1% mean abundance) could be assigned to 49 families or to a higher taxonomic level, if the assignment to the family level was not possible (Fig. 2). Within this group of most abundant ASVs, the majority belonged to the phyla Actinobacteria (75 ASVs) and Proteobacteria (29 ASVs), followed by Firmicutes (17 ASVs) and Chloroflexi (14 ASVs).

A few families/groups occur in more or less strong dominance along the entire moisture transect, such as *Acidimicrobiia*, *Frankiales* (*e.g.*, *Blastococcus*), *Solirubrobacteriaceae* (*e.g., Conexibacter*, *Solirubrobacter*), the lineage AKIW781 of *Chloroflexi* and the *Burckholderiaceae* (*e.g., Acidovorax*, *Pelomonas*, *Ralstonia*). Other families occur very prominently only at individual sites, such as *Halomicrobiaceae* (*e.g.*, *Natronomonas*, *Halomicrobium*) at a 20–30 cm depth in CS, *Bacillaceae* (*e.g.*, *Aquibacillus*) at a 20–30 cm depth in AL, or *Xanthomonadaceae* (*e.g.*, *Stenotrophomonas*, *Xanthomonas*) at a 0–5 cm depth in AL. Overall, almost all of these groups show a relatively high abundance in both DNA pools.

### Differences in the structure of the microbial communities in the e- and iDNA pools

**Fig 3:**
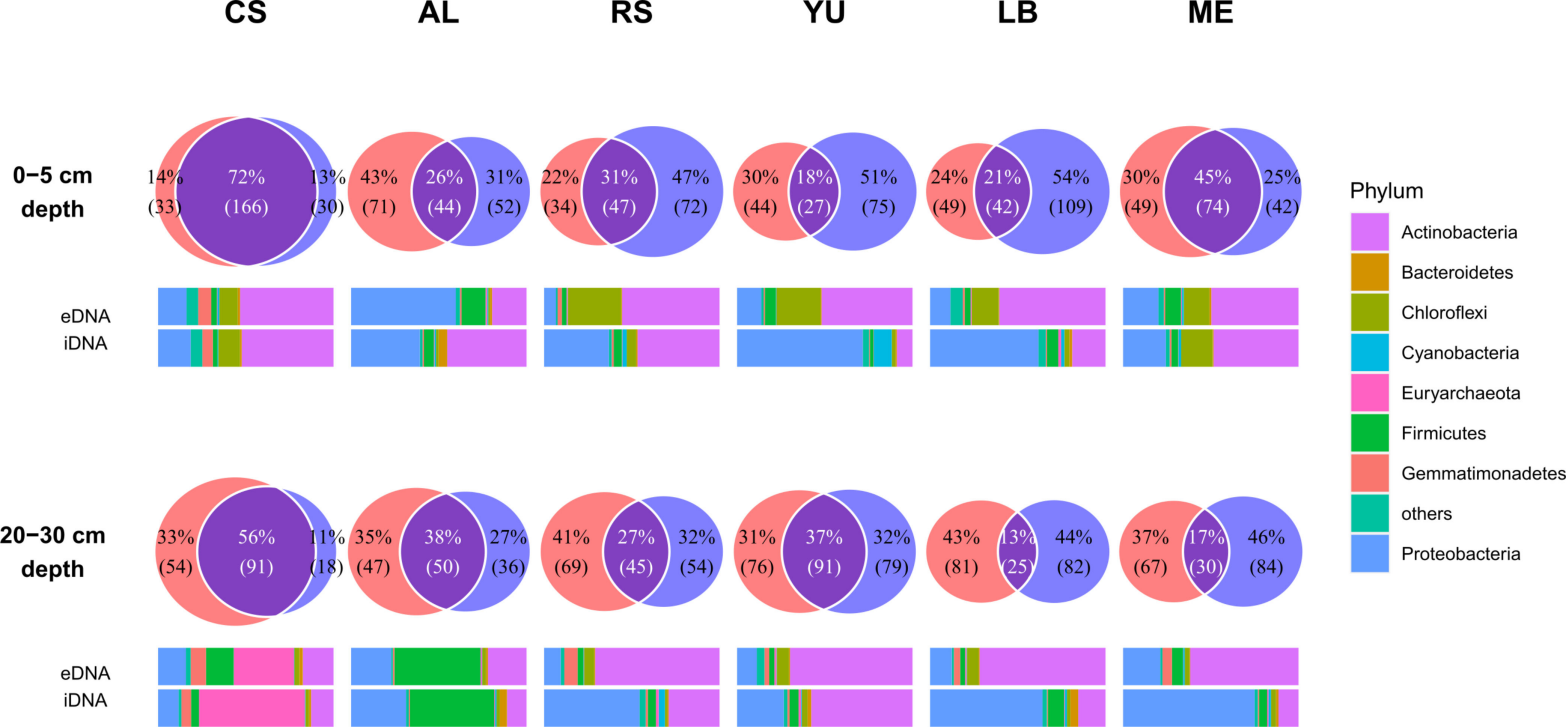
Microbial community structure and relationship between eDNA and iDNA pools at six soil sampling sites in the Atacama Desert: CS, AL, RS, YU, LB, and ME. Venn diagrams (each upper row) of eDNA (red bubble) and iDNA (blue bubble) ASV intersections (violet area) for samples collected at 0–5 and 20–30 cm soil depth. Numbers indicating the numbers of different ASVs (replicates pooled by mean first) refer to relative abundances of reads unique to eDNA or iDNA. Bars (each lower row) show relative abundances of main phyla in the eDNA and iDNA pools at the indicated sampling depth. Low abundance ASVs (<0.1%) were not counted.

The separation of iDNA from eDNA showed clear differences between the two pools (*P* < 0.001) with varying proportions of unique and shared (overlap) ASVs (Fig. 3). With the largest overlap of shared ASVs in both soil depths (72.5% in 0–5 cm and 55.8% in 20–30 cm), CS differs from all other study sites along the transect (*P* < 0.001, CS vs other sites), especially the CS surface sample, which showed only a smaller amount of unique ASVs in both pools (14.4% eDNA and 13.1% iDNA). In contrast to CS, all other sites are characterized by a lower proportion of shared ASVs, ranging between 18.5% to 44.8% in 0–5 cm depth and 13.3% to 37.6% in 20–30 cm depth. Accordingly, a higher proportion of unique ASVs was found in both DNA pools. A significant trend between both soil depths was not observed.

The differences observed in the two DNA pools and between CS and the other locations in terms of ASV proportions were also reflected in the structure and the relative abundances of the microbial communities (Fig. 3; Fig. S2). The surface sample of CS showed about the same distribution of individual phyla in both DNA pools (dominated by Actinobacteria and Proteobacteria), reflecting the high proportion of shared ASVs. A similar trend in the structure and relative abundance of the microbial communities in both DNA pools can also be seen in the surface sample from ME (also dominated by Actinobacteria and Proteobacteria, but with a high proportion of Cyanobacteria and Chloroflexi) and the samples from the 20–30 cm soil depth from CS (dominated by Euryarchaeota) and AL (dominated by Firmicutes). In all other study sites and sampling depths, the microbial community composition differed notably between the iDNA and eDNA pools. For example, the eDNA pools in YU and in LB (both at 0–5 cm soil depth) were dominated by Proteobacteria (71% and 61%, respectively), while the iDNA of the surface layers was dominated by Actinobacteria (51% and 60%, respectively).

Besides these shifts in abundance, there were also some taxa that were unique to either the eDNA or iDNA pool (Fig. 3). The most dominant groups in the eDNA pool were *Acinetobacter*, *Burckholderiaceae*, *Curvibacter*, *Methylosinus*, *Micrococcus*, *Pseudomonas*, and *Sericytochromatia*, and for the iDNA pool Acidimicrobia, the lineage AKIW781 of Chloroflexi, *Frankiales*, *Longimicrobiaceae*, *Rubrobacter*, and *Solirubrobacteriaceae*. These unique ASVs contributed to the differences in the community structure between the eDNA and iDNA pools described above due to their membership of the dominant phyla, such as Actinobacteria and Proteobacteria.

**Fig 4:**
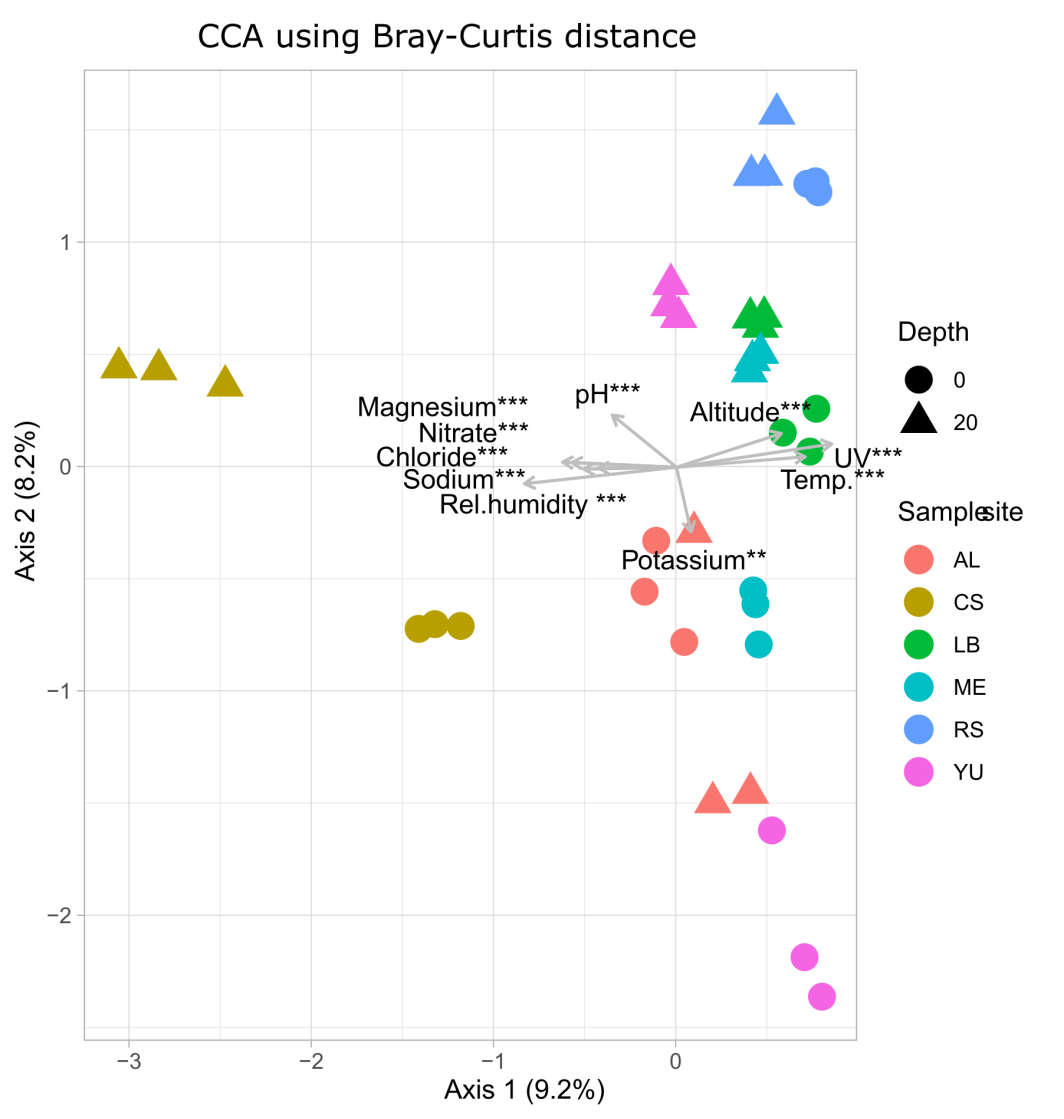
Canonical correspondence analysis (CCA) of the microbial community for the iDNA pool based on Bray–Curtis dissimilarities. In total, CA 1 and CA 2 explained 17.5% of the variance between samples from the different study sites along the transect (CS = Coastal Sand, AL = Aluvial Fen, RS = Red Sand, YU = Yungay, ME = Maria Elena, LB = Lomas Bajas). Environmental variables are projected as black vectors.

The relationship of ASV (iDNA pool) distribution and environmental parameters (Table S4) was examined by applying a CCA (Fig. 4; Table S5). Even if the adjusted explained compositional variation was low (17.5%), some parameters, such as chloride (*P* < 0.001), magnesium (*P* < 0.001), potassium (*P* < 0.011), pH (*P* < 0.001), UV (*P* < 0.001), elevation (*P* < 0.001), air temperature (*P* < 0.001), and relative humidity (*P* < 0.001) showed a significant impact on the ASVs distribution. A strong correlation was found between the unique community of CS and the relative humidity as well as the chloride and magnesium content, which clearly separated this site from all other sides along the transect. All other study sites were mainly influenced by air temperature, UV, and elevation. When comparing the differences in the community composition of the sample site (depth 0 and 20 cm combined), all sites differ (Table S6).

**Fig 5:**
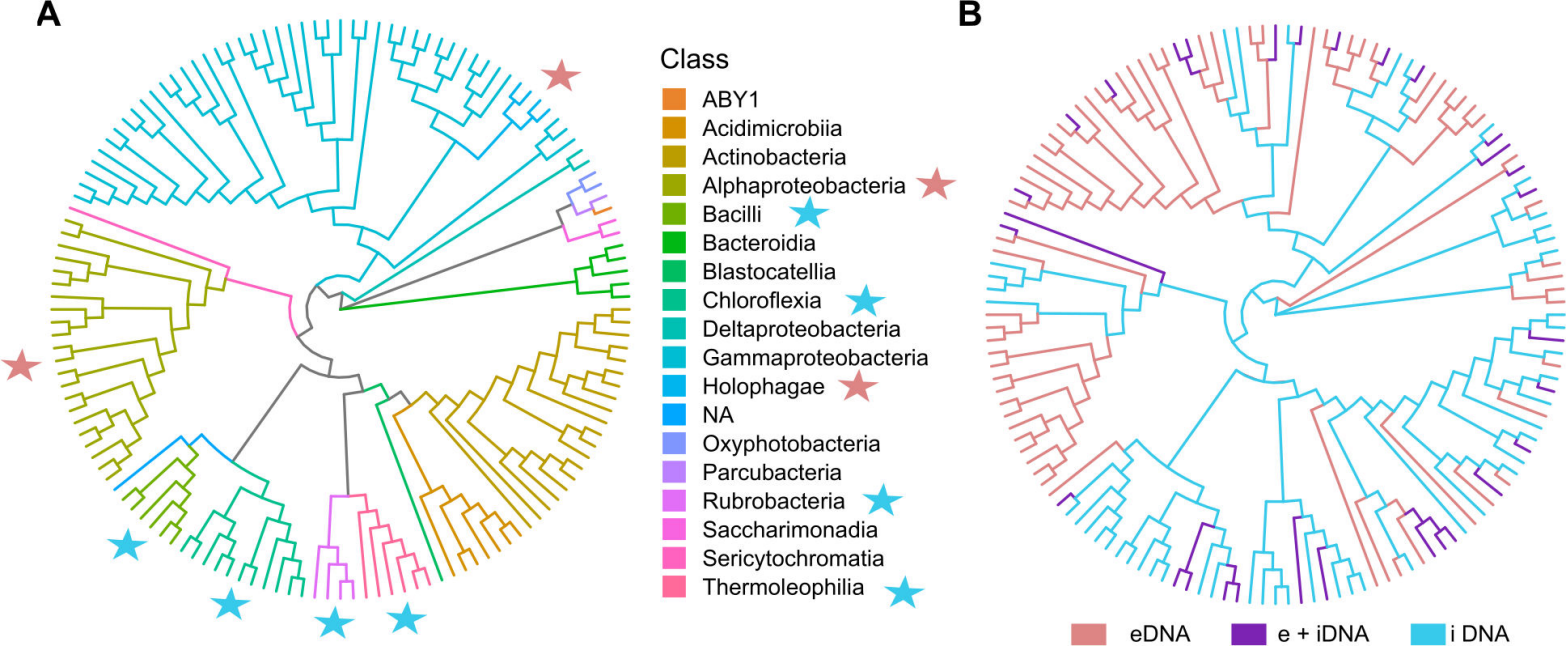
(A and B) Circular phylogenetic tree of the microbial community composition exemplarily for the surface layer (0–5 cm depth) in Yungay (0.1% abundance); color codes reflect microbial classes (**A**). Clades unique to the e- or iDNA are shown in (**B**). Additionally, red and blue stars indicated predominate eDNA and iDNA clades in (**A**). NA means no taxonomic class.

In some cases, the differences between the communities of eDNA and iDNA were extreme (Fig. 5A and B). As shown already in Fig. 3, there is only a small overlap of eDNA and iDNA ASVs, and many ASVs are unique in each pool (Fig. 5B). Some groups of ASV, *e.g.* almost all Bacilli, Chloroflexia, Rubrobacteria, and Thermoleophilia were only present in the iDNA (see Fig. 5A and B, blue stars), while other groups (*e.g.* Alphaproteobacteria and Holophagae, red stars) only exist in the eDNA pool.

### Microbial generalists and specialists along the moisture transect

In order to analyze the microbial communities along the transect and their significance in each study site, an analysis of the iDNA pool was carried out on generalists and specialists. Specialist ASVs were detected using the indicator-value analysis and predominately occur in distinct samples. Generalists are defined as ASV that occur with >0.1% abundance in 90% of our samples.

Generalists occurred at every study site and depth, while specialists were not detected at CS in the top soil or at ME. Bacterial generalists comprise four phyla with 16 ASVs, which were assigned to Actinobacteria, Chloroflexi, Firmicutes, and Proteobacteria (Fig. S3). Actinobacteria and Proteobacteria were the most dominant phyla, each with seven related ASVs. As *Actinobacteria*-related groups *Acidimicrobiia*, *Frankiales* and *Geodermatophilaceae* were identified as well as *Burkholderiaceae*, *Methylophilaceae*, and *Xanthomonadaceae* for the Proteobacteria-related groups. While the *Proteobacteria*-related ASVs are about evenly distributed across the transect, with the exception of *Xanthomonadaceae*, which predominated in the surface sample of AL, the *Actinobacteria*-related ASVs tend to show a dominance for certain locations. In the latter phylum, ASV2 is somewhat more evenly distributed, while all other ASVs show a clear preference for a specific site [*e.g.*, ASV9 dominated at RS (0–5 cm), ASV11 at all depths of LB, ASV18 at YU (0–5 cm), and ASV15 at ME (0–5 cm)]. The AKIW781-related ASV10 predominate in the surface layer of YU and ME, and the *Alicyclobacillaceae*-related ASV3 (Firmicutes) in AL (20–30 cm). All 16 ASVs of the generalists belonged to the top 50 ASVs.

In total, 40 ASVs were identified as habitat specialists, which were assigned to seven phyla, of which Actinobacteria (12 ASVs), Chloroflexi (8 ASVs), Euryarchaeota (8 ASVs), and Firmicutes (6 ASVs) were the dominant groups (Fig. S4). The specialists were dominant (indicator value >0.8) in only one study site along the transect. The highest proportions of Actinobacteria-related specialists were detected in the subsoil samples of the hyperarid sites RS, YU, and LB (with the exception of ASV28, which was dominant in the surface sample). A similar pattern is visible in the Chloroflexi-related ASVs (mainly lineage AKIW781), but in contrast to the Actinobacteria-related specialists, these taxa were mainly found in the surface layer of the respective study sites (RS, YU, and LB). All Euryarchaeota-related ASVs (mainly *Halomicrobiaceae*) were dominant in the subsurface layer of CS, and the *Firmicutes*-related specialists (mainly *Bacillaceae*) were detected in the subsurface samples in CS and AL. In general, no predominant habitat specialists could be identified in the soil surface at CS or for either soil depth intervals at ME. However, the importance of the habitat specialists for the Atacama Desert environment is demonstrated by the fact that 11 out of 40 ASVs belong to the top 50 ASVs.

## DISCUSSION

### Findings from the eDNA and iDNA differentiation

The analysis of the microbial communities’ abundance and diversity in natural environments is usually based on the total DNA extracted from environmental samples. No distinction is made between DNA from intact living cells (iDNA) and DNA from dead cells predominantly preserved “naked” in the sediment (eDNA). In environments that support active microbial life with high turnover rates, such as grass- or farmland, the eDNA pool is continually replenished by the biomass turnover of living cells, resulting in e- and iDNA pools being similar in terms of community composition (29). In contrast, extreme habitats, such as the Atacama Desert (Fig. 1), are characterized by microbial communities that are only sporadically active or have only low rates of metabolism (14). For this reason, methods based on mRNA often fail because of the low RNA level in desert soils. Our results show that the microbial communities represented by the e- and iDNA pools differ clearly in the Atacama Desert (Fig. 3 and 5; Tables S6 to S8), and that considering both pools separately is essential for understanding microbial life in these extreme habitats.

According to Torti *et al*. (30), the iDNA pool can theoretically include not only DNA from intact living cells, but also DNA from already dead, but structurally intact cells. The death and lysis of microbial cells are time-dependent processes that are controversial with regard to the question “How dead is dead?” (31). Cell lysis is dependent on the type of organism, the lethal agent and the cell damage, and in most cases leads to cell disintegration after minutes to hours (32). In our study, we define the iDNA pool as the fraction of microbial communities representing intact, living (supported by growth tests and live dead staining method; Fig. S6; Table S9), and potentially active microorganisms. This also applies to endospores, which end up in the iDNA pool during our separation process. In contrast to the DNA from the intact cells (the actual iDNA pool), the spore DNA is not extracted from the endospores by the mild extraction procedure used (33). This assumption aligns with the phylogenetic analyses of the iDNA sequences, which do not correspond to the presence of endospores from the Firmicutes phylum at our study sites (Fig. 2), despite Firmicutes being known as spore-forming bacteria (14). The living microbial community can only be considered in detail, if the eDNA pool is separated. Extracellular DNA could escape degradation processes *via* adsorption mechanisms and accumulate in sediments (34), which in turn cannot be imaged using conventional total DNA extraction methods. The eDNA might also include DNA material from cell lysis, active secretion by living cells, or allochthonous input of biogenic matter (35–37). In contrast to free eDNA, which is degraded within a few days, adsorbed DNA on minerals can remain in sediment for a long and even geological period of time (38, 39). Clay minerals for instance could enhance the DNA preservation through adsorption onto the mineral surface (40, 41). Adsorbed DNA can also be transported spatially and temporally by sediment processes. For this reason, when estimating the present biodiversity, it is important to estimate eDNA and iDNA to discriminate from allochthonous or past microbial input (42).

In the Atacama Desert, where most investigated habitats are deficient in organic carbon and water, active release of DNA into the environment is unlikely. The amount of shared ASVs of e- and iDNA is highest in the coastal study area CS (Fig. 3) where fog and sea spray provide regularly moisture (Fig. 4; Table S4). The increased availability of water and nutrients enables active cell turnover, especially in the top soil of CS. In contrast, the set of shared ASVs decreased in arid and hyperarid regions, likely indicating less cell turnover. Generally, microbial metabolism is thought to be negligible for most of the time in these extreme soil environments (14, 18).

The quantification of eDNA using qPCR often failed for these samples due to the lower gene copy numbers (Fig. 1B). With one exception (location CS), gene copy numbers of eDNA were lower or not measurable compared with the iDNA. These data show that even in the hyperarid areas, an abundant living microbial community existed, apparently very well adapted to the hostile conditions of the desert. A series of phospholipid fatty acids confirmed the existence of bacterial life in these soils as a biomarker, since intact membrane phospholipids are only stable in living organisms and rapidly degrade after cell death (43, 44). Gene copy numbers of iDNA were supported by the quantification of PLFAs which are both in the same range (Fig. 1B).

The cladogram (circular family tree, Fig. 5) illustrates the importance of distinguishing between e- and iDNA. Some clades appear almost exclusively as eDNA (such as *Alphaproteobacteria* and *Holophagae*). If there is no differentiation between e- and iDNA, the no longer living organisms cannot be adequately considered.

Considering the shared pool of e- and iDNA (Fig. 3 and 5), we get a broader understanding of the viable and potentially active microbial communities (14, 36), since the identification of e- and iDNA of similar source can show the active cell turnover of the stationary phase (24). Based on this assumption, a putatively active community of Actinobacteria (mainly *Acidimicrobiia*, *Geodermatophilaceae*, *Frankiales*, *Rubrobacteriaceae* and *Solirubrobacteriaceae*) and Proteobacteria (primarily *Burkholderiaceae*, *Methylophilaceae*, *Moraxellaceae*, *Sphingomonadacea* and *Xanthomonadaceae*) dominated at all study sites (Fig. 2). Furthermore, another probably active taxon is the AKIW781 lineage, which dominated the phylum Chloroflexi, as well as *Alicyclobacillaceae* and *Bacillaceae* of the phylum Firmicutes. These spore-forming Firmicutes (45, 46) are well adapted to extreme conditions, such as drought or high UV radiation (47). A richness of endospore DNA in the iDNA pool can be excluded because the procedure to extract iDNA does not extract DNA from spores (14, 24). In addition, AKIW781 has been reported from desert soils and in lithic communities (48, 49). The presence of *Geodermatophilaceae* on altered rock surfaces and dry soils was described by Urzi *et al.* (50) and Normand (51). They are resistant to environmental stress, such as desiccation, high salinity or UV radiation. *Gammaproteobacteria*, within the families of *Xanthomonadaceae*, *Burkholderiaceae,* and *Moraxellaceae,* were also reported to survive under low nutrient availability and were found in rocks (52–55). The presence of lithobiotic microorganisms at all investigated study sites suggests a wide distribution of these microorganisms in arid environments preferring retreating niches, such as shaded rock surfaces (56). Colonizing refuge niches can be a strategy to reduce environmental stress, while niches form a protective environment by shielding against radiation, desiccation, and large temperature fluctuations (57).

### Insights into the living microbial community

To gain a more detailed insight into the living microbial community, we analyzed the composition of the iDNA in detail. Therefore, the differentiation between habitat specialist and generalist will help identify specific aspects on adaptation strategies and ways of living. The distinction between specialists and generalists can help to deepen the understanding of microbial diversity and its role in different ecosystems. Generalists indicate a broad environmental tolerance and are found in many habitats, while specialists show narrow environmental tolerance and live in a limited habitat (28, 58, 59).

#### Dominant phyla as generalists and their adaptation strategies

In general, the microbial communities were dominated by Actinobacteria and Proteobacteria followed by Firmicutes and Chloroflexi (Fig. 2 and 3), which were also identified in the iDNA pool as generalists (Fig. S3). These phyla consist of species that are highly adapted to extreme environmental conditions [*e.g.*, dryness or UV radiation (Fig. S5)]. Actinobacteria are pioneers for the colonization of initial soil ecosystems and therefore play an important role in the development of soils and the underlying biogeochemical cycles (60). Most identified Actinobacteria belong to the class *Acidimicrobiia* and to the nitrogen-fixing bacteria *Frankiales* (61). Both were previously described as members of the core actinobacterial microbiome in low-elevation Atacama soils (62, 63). Genome-resolved analyses indicate *Acidimicrobiia* are metabolically flexible, possessing the ability to use atmospheric H_2_ to support aerobic respiration and carbon fixation (64). Together with *Geodermatophilaceae* and the Chloroflexi lineage AKIW781, they occurred in high abundance in RS, YU, LB, and ME (Fig. 2). Moreover, *Alicyclobacillaceae* (Firmicutes) and *Burkholderiaceae* (Proteobacteria) occurred in high abundance in all study sites. *Burkholderiaceae* can inhabit diverse ecological niches due to their capability to perform different metabolic functions (65). They are found in soils, ground water, and rock surfaces (49). *Burkholderiaceae* were described to promote plant growth by enhancing nitrogen fixation and improving overall host adaptation to environmental stresses (65, 66).

The widespread presence of generalists indicates their tolerance to different soil characteristics, competitive ability, and widespread use of resources (67, 68). Compared with specialists, generalists can react quickly to the availability of resources, and they are less controlled by environmental selection than specialists (69). The high abundance of eDNA and iDNA for *Acidimicrobiia*, *Geodermatophilaceae*, *Frankiales,* and *Burkholderiaceae*, and their occurrence as generalists, indicates active cell turnover in the Atacama Desert, which is also supported by the findings of Schulze-Makuch *et al.* (14). We see the occurrence of hypolithic microorganisms, such as *Burkholderiaceae*, or *Geodermatophilaceae*, and their potential in terrestrial colonization in the Atacama Desert. Mineral weathering can be a microbial-driven process, which is important for soil formation as well as nutrient supply and by this for the general developments of the habitats. Based on the community description, it could be shown that biological nitrogen fixation can be potentially carried out by microorganisms, such as *Frankiales*. This is a key process in the nitrogen cycle and the main source of nitrogen available in soil. Other generalists present, such as *Solirubrobacterales*, can handle the lowest soil organic carbon levels (70). In addition to the previously mentioned high-abundant generalists, *Acidimicrobiia* refer to a potentially active community indicating in the Atacama Desert. Together with *Rubrobacteriaceaea*, *Acidimicrobiia* are capable of hydrogenotrophic carbon fixation (64), enabling soil formation processes.

#### Specialized microorganisms across study sites

The differences in the microbial communities of the various study sites are more pronounced (Fig. 4), with an overabundance of specialists compared with generalists (Fig. S4). Changes within the community are evident along the transect when going from wetter to drier conditions, as changes between the two investigated depths. The abundance of halophilic archaea (*Haloadaptaceae*, *Halobacteriaceae*, *Halomicrobiaceae*, and *Halobacteriales*) at a depth of 20 to 30 cm at the CS site is remarkable. The deep layers of CS were enriched in sodium, chloride, and sulfate (Fig. S4; Table S5), a necessity for halophilic and halotolerant microbes. Furthermore, the availability of nitrate indicates growth through denitrification *via* nitrate reduction (71). The presence of *Natronomonas*, member of *Halobacteriaceae*, is mainly driven by high nitrate, chloride, sodium and magnesium concentrations in the deep layers of CS (Fig. S4; Table S5). *Natronomonas* is an extremely haloalkaliphilic archaeon formerly isolated from alkaline brines that are enriched in carbonate and chloride. They can tolerate extreme saline conditions, high pH (~11), and are able to assimilate nitrate (72). In addition, it was shown that halophilic archaea occur in both the eDNA and iDNA pool (Fig. 3), indicating that there is a steady cell turnover. The coastal sea spray is the main source of chloride and a valuable amount of sodium, making CS a unique habitat compared with the Atacama’s hyperarid regions. Members of Firmicutes (*Bacillaceae*, *Paenibacillaceae*, *Sporolactobacillaeae*) and Proteobacteria (*Geminicoccaceae*) occurred in high abundance at a depth of 20 to 30 cm, which could not be observed at the surface of CS. *Geminicoccaceae* has been isolated from desert sandy soils and can adapt to environmental stress, such as desiccation, osmotic stress, or carbon starvation (73).

Although specialists can be found at certain depths, they are not necessarily tied to the location, but are adapted to the corresponding soil conditions. The specialists in AL also occur with high frequency at the 20–30 cm depth and consist mainly of *Bacillaceae*. The surface of RS was characterized by high abundance of Chloroflexi, such as lineage AKIW781 and *Kallotenuaceae*. In contrast, these families are less abundant in the deeper layers of RS, while *Euzebyaceae* (class *Nitriliruptoria*), *Micromonosporaceae*, and *Solirubrobacteraceae*, all belonging to Actinobacteria, were found in higher abundance. *Nitriliruptoria* were detected by Idris *et al.* (62) as rare actinobacterial taxa in the Atacama region. In YU, specialists, such as *Solirubrobacteraceae* and AKIW781 dominated, while at 20–30 cm Acidobacteria Subgroup 6, *Gaiellales* and IMCC26256 were more common. The deeper soils were characterized by higher water content (12%, Table S4) and high sulfate concentration. *Gaeiellales* have been previously obtained from diverse environments, such as saline-alkali soils (74), mangrove wetlands (75), lithic environments (76), and marine deep-sea sediments (77). In LB, *Sphingomonadaceae* and JG30-KF-CM45 occurred in high abundance in the top soils, whereas *Euzebyaceae* and *Solirubrobacteraceae* could be identified as specialist in the deeper soils. *Solirubrobacterales* have the ability for chemosynthetic CO_2_ fixation (78), promote soil organic matter and enhance soil fertility (79). Their abundance correlated to the lower temperatures and higher relative humidity at CS compared to other regions.

Recent studies show that differences in available moisture for extremely hyperarid soil microbial communities can have impacts on community composition, functional potential, and the capacity of growth in response to soil wetting (80). Nevertheless, the microbial profiles can provide an indication of supposed life and adaptation strategies in hyperarid areas. The analysis of the specialists shows that every niche in the Atacama Desert is colonized by specialized well adapted microorganisms, thus enabling active life in an otherwise hostile environment. Differences in the living microbial community can be observed due to differentiation in e- and iDNA. Combined with the distinction between generalists and specialists, the diversity of a living community in nutrient-poor and water-limited areas like the Atacama Desert can now be understood in more detail.

### Conclusion

We conducted a soil microbial study using e- and iDNA extraction methods combined with NGS technology and analysis tools to study bacterial communities inhabiting Atacama Desert soils. Our goal was to go beyond descriptions of the composition of the entire microbial community in such a low-biomass environment. By distinguishing between e- and iDNA, it was possible to focus on the living community at the cell extraction level. In low-biomass habitats, this is an essential step in the analysis of the initial microbial community. Starting from the iDNA, we were able to look deeper into the living community and identify habitat-specific microorganisms. Differences in the sites, especially with regard to water availability, become visible when focusing on iDNA only. Cell turnover appeared to be noticeably reduced in the drier areas, making analysis at the mRNA or protein level challenging if not impossible. The distinction between specialists and generalists in the iDNA pool allowed a detailed understanding of the composition of the living microbial community. Specialists were adapted to the limited resources, occurred in more inconsistently distributed habitats, and had smaller overall population sizes than generalists. Because of their resource specialization, suitable habitats for specialists are spatially limited. For example, we found that the location affected by the salt-rich coastal sea spray harbored halophilic archaea as specialists. Furthermore, we observed viable microorganisms (such as *Acidimicrobiia*, *Geodermatophilaceae*, *Frankiales*, and *Burkholderiaceae*) as generalists, known for involvement in initial soil formation processes, such as carbon and nitrogen fixation as well as mineral-weathering processes.

## MATERIALS AND METHODS

### Field site description and sampling

Soil samples were collected from the surface (0–5 cm) and near-subsurface (20–30 cm) at six locations along a west–east transect with a decreasing moisture gradient (Fig. 1A). Samples were collected in sterile sampling containers using appropriate tools (*e.g.*, spoons sterilized with ethanol) and latex sampling gloves. The most western and least arid sampling location was designated Coastal Soil (**CS**, 481 masl, S 23.8247, W 70.4888) and consisted of widely spaced vegetation and distinct hypolithic communities. The upper 20 to 30 cm consisted of silty-to-sandy material with occasional large weathered clasts. No vegetation was present anymore at the next sampling site further east, which was designated Alluvial Fan (**AL**, 847 masl., S 24.0008, W 70.2958). The surface of the alluvial fan is covered with loose sandy sediments, with some clasts reaching up to 15 cm in diameter. Below this top layer lies a few-centimeter thick spongy evaporitic layer and variable alluvial deposits. The next site along the transect is part of the hyperarid region and was designated Red Sands (**RS**, 1027 masl, S 24.10007, W 70.1288). The top layer is a few-centimeter thick and consists of cemented desert crust, while the sediments beneath are composed of poorly sorted sand with crude lamination. The next site along the transect is Yungay (**YU**, 1004 masl, S 24.0883, W 69.9946) being the most lithological diverse site. Lithic particles on the surface range from silt to pebbles, and between them occur partially cracked, cake-shaped sulfate mineral bearing lobes (losa). Beneath the surficial layer is poorly sorted sand in some areas, while in other areas there is a highly porous sponge-like gypsum horizon. The reference site Lomas Bayas (**LB**, 1528 masl, S 23.3933, W 69.6039) is located in the northeast from the transect. LB is covered by angular clasts of up to 40 cm in diameter. Between the clasts the surface is largely covered by sandy to gravely material and the soil beneath is composed of clay to cobble-sized particles of angular shape with poor to no sorting. Marina Elena (**ME**, 1313 masl, S 22.2631, W 69.7243) is the northernmost study area. ME is a field of cobbles and boulders reaching a few meters in diameter. The top soil between the boulders consists of coarse sand to fine pebbles, while the sediments below are composed of weakly cemented silt to coarse sand with abundant planar and cross-bedding.

### Extraction of extracellular (e) and intracellular (i) DNA

DNA was extracted according to the method of Alawi *et al.* (81), which was slightly modified as follows. All samples were processed in triplicates and included blank controls for the iDNA and eDNA.

#### Soil removal

In a 15 mL sterile conical tube, 6.5 g of Atacama Desert soil sample and 0.4 g polyvinylpolypyrrolidone (PVPP, Sigma-Aldrich order no. 77627) were carefully suspended in 5–6 mL of cold (4°C) sodium phosphate (NaP) buffer (Na_2_HPO_4_ and NaH_2_PO_4_, 0.12 M, pH 8) to form a slightly viscous slurry [Ogram *et al.* (40)]. The tube was chilled on ice for 1 min and then shaken twice for 5 min at 150 rpm in a horizontal position on an orbital shaker with cooling on ice in between for 3 min. After a centrifugation step (500 × *g*, 4°C, 10 min, swing-out rotor), the supernatant was transferred to a sterile 15 mL conical tube and kept on ice. The remaining pellet was resuspended in 3.0–3.5 mL NaP buffer, and the separation procedure was repeated three more times with all supernatants pooled in one tube (6–8 mL).

#### eDNA and iDNA separation

After centrifugation (4,643 × *g*, 4°C, 1 h, swing-out rotor), the supernatant representing the eDNA fraction was transferred to a new tube, and the pellet containing the intact cells with the iDNA were kept on ice. The supernatant was passed through a 0.2 µm syringe filter (VWR international, cellulose acetate) pre-rinsed with 500 µL NaP buffer. After passing the sample, the filter was rinsed with another 500 µL NaP buffer and then added to the filtered eDNA solution. The filtrate was collected in a sterile 50 mL conical tube and kept on ice until further processing. The iDNA containing pellet was carefully resuspended in 1 mL NaP buffer, transferred to a sterile 2 mL reaction vial (Eppendorf, low binding), and centrifuged for 20 min at 12,000 × *g* to remove any remaining eDNA.

#### iDNA extraction

The supernatant was discarded, and the pellet suspended in 750 µL NaP buffer by incubation in a thermal shaker for two periods of 5 min at 70°C and 250 rpm with cooling on ice in between for 2 min. The suspension was transferred to a PowerBead tube (Mo Bio Laboratories, Inc.) (buffer removed), mixed with 60 µL solution C1 (PowerSoil Kit, Mo Bio) and vortexed horizontally for 10 min according to the kit instructions. After centrifugation (10,000 × *g*, 1 min), the supernatant was transferred to a new low binding 2 mL reaction vial and mixed with 250 µL solution C2 (vortex 5 s; incubation 5 min, 4°C; centrifugation at 10,000 × *g*, 1 min). The supernatant (ca. 1 mL) was kept on ice in a 50 mL sterile conical tube.

#### Further eDNA and iDNA handling

iDNA and eDNA solutions were each mixed with the threefold amount of guanidine hydrochloride (GuaHCl) (6M GuaHCl in TE buffer, pH 6.7, 10 mM Tris HCl, 1 mM EDTA) and 15 µL (iDNA) or 18 µL (eDNA) silica suspension [prepared according to reference (81)] by inverting the tubes several times. The tubes were secured horizontally on an orbital shaker and shaken for 45 min and 175 rpm to bind the DNA to the silica particles. Finally, the silica was allowed to settle for 10 min on ice before the centrifugation step (4,643 × *g*, 10 min, RT, swing-out rotor). The supernatants were carefully removed by suction except for a residual of 1.5 mL to resuspend the silica pellets. The suspensions were transferred to new sterile 2 mL low-binding reaction vials and centrifuged (9,000 × *g*, RT, 3 min). The supernatants were discarded, and the pellets were each washed with 600 µL washing buffer (55% EtOH, 70 mM NaCl, 10 mM Tris, 2.6 mM EDTA) (vortex 2s, centrifuge at 9,000 × *g*, 1 min). The resulting supernatants were discarded, the pellets were again centrifuged (9,000 × *g*, RT, 3 min), and the remaining buffer was removed. The pellets were air-dried in a clean bench for 15 min. Finally, the DNA was eluted by resuspending the pellets in 100 µL (eDNA) or 80 µL (iDNA) Tris buffer (1 mM Tris, pH 8.0, preheated to 50°C) by pipetting up and down, vortex 2s, followed by an incubation period of 10 min in a thermal shaker (50°C, 300 rpm). The silica suspensions were centrifuged (9,000 × *g*, RT, 2 min), and the supernatants were transferred to new 1.5 mL low-binding reaction vials. To remove any residual silica particles, the supernatants were centrifuged again (9,000 × *g*, RT, 5 min) and transferred to new vials. DNA concentrations were mostly below the detection limit of fluorometric quantification methods.

### Quantitative PCR analysis (qPCR)

qPCR was performed using a CFX Connect Real-Time PCR Detection System (Bio-Rad, CA, USA) in triplicates of iDNA and eDNA and corresponding blank controls using iTaq Universal SYBR Green Supermix (Bio-Rad). DNA was amplified with the universal primers 331F and 797R (8) and the following cycling parameters: initial denaturation at 95°C, 4 min followed by 40 cycles (95°C, 30 s; 58°C, 30 s; 72°C, 30 s; 80°C, 3 s). The correlation coefficient for the standard curves was 0.99, and the PCR efficiency was on average 90%. The standard was a known concentration of a 16S rRNA gene PCR fragment of *Bacillus subtilis*.

### 16S rRNA gene amplicon pool preparation for Illumina MiSeq sequencing

PCR amplification targeted the hypervariable region V4 of the 16S rRNA (forward primer, 515F: 5′-GTGCCAGCMGCCGCGGTAA-3′; reverse primer, 806R: 5′-GGACTACHVGGGTWTCTAAT-3′, each of them specified with 6 bp tags). PCR amplification was performed in at least triplicates in 25 μL reactions (2.5 µL 10× PCR buffer, 0.5 µL ultrapure dNTP-mix (5 mM), 0.25 µL of each primer (10 mM), 1.5 µL MgCl_2_ (25 mM), 2–5 μL template, 0.25 µL HotStar Taq polymerase (Qiagen, Hilden, Germany) under the following conditions: initial denaturation at 95°C for 15 min, followed by 10 cycles of 95°C for 30 s, 65°C–1°C/cycle for 30 s, 72°C for 45 s, 25 to 40 cycles (depending on DNA concentration of the different samples) of 95°C for 30 s, 56°C for 30 s, 72°C for 45 s, and a final extension step of 10 min at 72°C. The reactions were pooled, purified with Agencourt AMPure XP magnetic beads (Beckman Coulter, CA, USA), and quantified with the Qubit Fluorometer (Invitrogen™, Thermo Fisher Scientific, USA). Purified PCR amplicons from all samples were pooled in equimolar ratios to a final concentration of approximately 120 ng/µL. Library preparation and sequencing of the amplicon pool with the Illumina MiSeq technology were done by Eurofins Genomics (Ebersberg, Germany).

### Processing of 16s rRNA MiSeq data

Paired-end sequencing raw reads were demultiplexed and quality trimmed using Cutadapt v2.5 (82), discarding low-quality bases (-q 20) and short reads (-m 150). DADA2 v1.10.2 (83) was used to generate an ASV table with pooling approach and assign taxonomy, including forward and reverse read merging. Non-default parameter for the different functions were the following: filterAndTrim: truncLen 240/200, minLen 200, maxN 0, maxEE 2,2, truncQ 2, rm.phix TRUE; dada: pool TRUE; removeBimeraDenovo: method consensus. For taxonomic assignment SILVA database v138 (84) was used.

### Statistical analysis, visualization, generalists, and specialists

For statistical analysis and visualization, R v3.6.1 (85) including package ggplot2 v3.2.1 (86), phyloseq v1.28.0 (87), vegan v2.5–6 (https://cran.r-project.org/package=vegan), labdsv 2.0-1 (https://cran.r-project.org/package=labdsv), igraph v1.2.4.1 (https://cran.r-project.org/package=igraph), and VennDiagram v1.7.3 (https://CRAN.R-project.org/package=VennDiagram),was used. For ordination, pyhloseq with CCA method and Bray–Curtis distance was used. Environmental parameters were fitted using the vegan envfit function. Replicates were summed by (arthicmetic) mean. Community differences were tested using PERMANOVA and the adonis2 function of the vegan package. Venn diagrams were generated using the VennDiagramm package and using ASV’s > 0.1% relative abundance. Specialists were detected using replicates of iDNA samples of depth 0–5 cm and 0–20 cm and the indicator species detection (88) implemented in indval function of the labdsv package using *P* ≤ 0.05 and indicator values ≥0.8. We defined generalist as a non-specialist (ASVs not detected as specialist) that showed an abundance of >0.1% in 90% of the samples of the iDNA sample of depth 0–5 and 0–20 cm

### Phospholipid fatty acid (PLFA) analysis

This analysis was carried out by MicrobialInsights (Knoxville, Tennessee, USA) using the following procedure. About 60 g of freeze-dried and ground sample material was extracted using a flow blending system with a methanol (MeOH)/dichloromethane (DCM)/ammonium acetate (NH_4_OAc) buffer (2:1:0.8, pH 7.6) solvent mixture following a modified Bligh and Dyer method (89). After transferring the extract into a separation funnel, 50 µg of 1-myristyl-(D_27_)-2-hydroxy-sn-glycerol-3-phosphocholine was added as internal standard for subsequent quantification. To separate the water from the organic phase, DCM and water were added, at a ratio of 1:1:0.9. Subsequently, the organic phase was removed, and the water phase was re-extracted twice with DCM. Finally, all organic phases were combined. The organic extract was then separated into different fractions of polarity [low polar lipids, free fatty acids, glycolipids, and phospholipids (PL)] following the column separation method described in Zink and Mangelsdorf (89). To improve the recovery of the PLs, the column material was additionally rinsed with 25 mL of a MeOH/water (60:40) mixture. After phase separation by adding 15 mL of DCM and 3.5 mL of water (MeOH/DCM/water, 1:1:0.9), the organic phase was removed, and the water phase was re-extracted twice with DCM. All organic phases were combined and dried before storing the sample extracts at −20°C until analysis. To obtain the PLFAs, half of the PL-fraction was exposed to an ester-cleavage procedure outlined by (90). Afterwards, the resulting PLFAs were detected using gas chromatography–mass spectrometry (GC-MS). The measurement was conducted on a Trace GC Ultra coupled to a DSQ MS (both Thermo Electron Corporation). The GC was equipped with a cold injection system operating in the splitless mode and a SGE BPX five fused-silica capillary column (50 m length, 0.22 mm ID, 0.25 µm film thickness) using the following temperature conditions: initial temperature 50°C (1 min isothermal), heating rate 3°C/min to 310°C, held isothermally for 30 min. Helium was used as carrier gas with a constant flow of 1 mL/min. The injector temperature was programmed from 50 to 300°C at a rate of 10°C/s. The MS operated in the electron impact mode at 70 eV. Full-scan mass spectra were recorded from *m/z* 50–650 at a scan rate of 1.5 scans/s. Total amounts of PLFAs in pmol per gram soil dry weight were multiplied by 20,000 cells/pmol following a conversion factor presented by reference (91).

## Data Availability

The 16S rRNA amplicon sequences reported in this paper have been deposited in the EMBL-EBI database (accession no. PRJEB20402 with the sample IDs ERS1666624–ERS1666714).

## SUPPLEMENTAL MATERIAL

Supplemental material aem.01443-24-s0001.docx Tables S1 to S9, Fig. S1 to S6, and supplemental methods.

ASM does not own the copyrights to Supplemental Material that may be linked to, or accessed through, an article. The authors have granted ASM a non-exclusive, world-wide license to publish the Supplemental Material files. Please contact the corresponding author directly for reuse.

## References

[1] McKay CP, Friedmann EI, Gómez-Silva B, Cáceres-Villanueva L, Andersen DT, Landheim R. 2003. Temperature and moisture conditions for life in the extreme arid region of the Atacama Desert: four years of observations including the El Niño of 1997–1998. Astrobiology 3:393–406. doi:10.1089/153110703769016460 14577886

[2] Dunai TJ, González López GA, Juez-Larré J. 2005. Oligocene–miocene age of aridity in the Atacama Desert revealed by exposure dating of erosion-sensitive landforms. Geol 33:321. doi:10.1130/G21184.1

[3] Hartley AJ, Chong G, Houston J, Mather AE. 2005. 150 million years of climatic stability: evidence from the Atacama Desert, northern Chile. JGS 162:421–424. doi:10.1144/0016-764904-071

[4] Houston J, Hartley AJ. 2003. The central Andean west‐slope rainshadow and its potential contribution to the origin of HYPER‐ARIDITY in the Atacama Desert. Intl J Climatol 23:1453–1464. doi:10.1002/joc.938

[5] Clarke JDA. 2006. Antiquity of aridity in the Chilean Atacama Desert. Geomorphology (Amst) 73:101–114. doi:10.1016/j.geomorph.2005.06.008

[6] Amundson R, Dietrich W, Bellugi D, Ewing S, Nishiizumi K, Chong G, Owen J, Finkel R, Heimsath A, Stewart B, Caffee M. 2012. Geomorphologic evidence for the late pliocene onset of hyperaridity in the Atacama Desert. Geol Soc Am Bull 124:1048–1070. doi:10.1130/B30445.1

[7] Ewing SA, Sutter B, Owen J, Nishiizumi K, Sharp W, Cliff SS, Perry K, Dietrich W, McKay CP, Amundson R. 2006. A threshold in soil formation at Earth’s arid–hyperarid transition. Geochim Cosmochim Acta 70:5293–5322. doi:10.1016/j.gca.2006.08.020

[8] Navarro-González R, Rainey FA, Molina P, Bagaley DR, Hollen BJ, de la Rosa J, Small AM, Quinn RC, Grunthaner FJ, Cáceres L, Gomez-Silva B, McKay CP. 2003. Mars-like soils in the Atacama Desert, Chile, and the dry limit of microbial life. Science 302:1018–1021. doi:10.1126/science.1089143 14605363

[9] Wierzchos J, Rios A, Ascaso C. 2012. Microorganisms in desert rocks: the edge of life on earth. Int Microbiol 15:173–183. 23844476 10.2436/20.1501.01.170

[10] Cáceres L, Gómez‐Silva B, Garró X, Rodríguez V, Monardes V, McKay CP. 2007. Relative humidity patterns and fog water precipitation in the Atacama Desert and biological implications. J Geophys Res 112. doi:10.1029/2006JG000344

[11] Rundel PW, Dillon MO, Palma B, Mooney HA, Gulmon SL, Ehleringer JR. 1991. The phytogeography and ecology of the Coastal Atacama and Peruvian Deserts. aliso 13:1–49. doi:10.5642/aliso.19911301.02

[12] Michalski G, Böhlke JK, Thiemens M. 2004. Long term atmospheric deposition as the source of nitrate and other salts in the Atacama Desert, Chile: new evidence from mass-independent oxygen isotopic compositions. Geochim Cosmochim Acta 68:4023–4038. doi:10.1016/j.gca.2004.04.009

[13] Cordero RR, Damiani A, Jorquera J, Sepúlveda E, Caballero M, Fernandez S, Feron S, Llanillo PJ, Carrasco J, Laroze D, Labbe F. 2018. Ultraviolet radiation in the Atacama Desert. Antonie Van Leeuwenhoek 111:1301–1313. doi:10.1007/s10482-018-1075-z 29605897

[14] Schulze-Makuch D, Wagner D, Kounaves SP, Mangelsdorf K, Devine KG, de Vera J-P, Schmitt-Kopplin P, Grossart H-P, Parro V, Kaupenjohann M, et al.. 2018. Transitory microbial habitat in the hyperarid Atacama Desert. Proc Natl Acad Sci U S A 115:2670–2675. doi:10.1073/pnas.1714341115 29483268 PMC5856521

[15] Connon SA, Lester ED, Shafaat HS, Obenhuber DC, Ponce A. 2007. Bacterial diversity in hyperarid Atacama Desert soils. J Geophys Res 112:G4. doi:10.1029/2006JG000311

[16] Bernhard N, Moskwa L-M, Schmidt K, Oeser RA, Aburto F, Bader MY, Baumann K, von Blanckenburg F, Boy J, van den Brink L, et al.. 2018. Pedogenic and microbial interrelations to regional climate and local topography: New insights from a climate gradient (arid to humid) along the Coastal Cordillera of Chile. CATENA 170:335–355. doi:10.1016/j.catena.2018.06.018

[17] Neilson JW, Quade J, Ortiz M, Nelson WM, Legatzki A, Tian F, LaComb M, Betancourt JL, Wing RA, Soderlund CA, Maier RM. 2012. Life at the hyperarid margin: novel bacterial diversity in arid soils of the Atacama Desert, Chile. Extremophiles 16:553–566. doi:10.1007/s00792-012-0454-z 22527047

[18] Crits-Christoph A, Robinson CK, Barnum T, Fricke WF, Davila AF, Jedynak B, McKay CP, Diruggiero J. 2013. Colonization patterns of soil microbial communities in the Atacama Desert. Microbiome 1:28. doi:10.1186/2049-2618-1-28 24451153 PMC3971613

[19] Jones RT, Robeson MS, Lauber CL, Hamady M, Knight R, Fierer N. 2009. A comprehensive survey of soil acidobacterial diversity using pyrosequencing and clone library analyses. ISME J 3:442–453. doi:10.1038/ismej.2008.127 19129864 PMC2997719

[20] Genderjahn S, Alawi M, Wagner D, Schüller I, Wanke A, Mangelsdorf K. 2018. Microbial community responses to modern environmental and past climatic conditions in Omongwa Pan, western Kalahari: a paired 16S rRNA gene profiling and lipid biomarker approach. JGR Biogeosciences 123:1333–1351. doi:10.1002/2017JG004098

[21] Schulze-Makuch D, Lipus D, Arens FL, Baqué M, Bornemann TLV, de Vera J-P, Flury M, Frösler J, Heinz J, Hwang Y, Kounaves SP, Mangelsdorf K, Meckenstock RU, Pannekens M, Probst AJ, Sáenz JS, Schirmack J, Schloter M, Schmitt-Kopplin P, Schneider B, Uhl J, Vestergaard G, Valenzuela B, Zamorano P, Wagner D. 2021. Microbial hotspots in lithic microhabitats inferred from DNA fractionation and metagenomics in the Atacama Desert. Microorganisms 9:1038. doi:10.3390/microorganisms9051038 34065975 PMC8151210

[22] Rissanen AJ, Kurhela E, Aho T, Oittinen T, Tiirola M. 2010. Storage of environmental samples for guaranteeing nucleic acid yields for molecular microbiological studies. Appl Microbiol Biotechnol 88:977–984. doi:10.1007/s00253-010-2838-2 20730531

[23] Tatangelo V, Franzetti A, Gandolfi I, Bestetti G, Ambrosini R. 2014. Effect of preservation method on the assessment of bacterial community structure in soil and water samples. FEMS Microbiol Lett 356:32–38. doi:10.1111/1574-6968.12475 24840085

[24] Genderjahn S, Lewin S, Horn F, Schleicher AM, Mangelsdorf K, Wagner D. 2021. Living lithic and sublithic bacterial communities in Namibian Drylands. Microorganisms 9:235. doi:10.3390/microorganisms9020235 33498742 PMC7911874

[25] Medina Caro D, Horstmann L, Ganzert L, Oses R, Friedl T, Wagner D. 2023. An improved method for intracellular DNA (iDNA) recovery from terrestrial environments. MicrobiologyOpen 12:e1369. doi:10.1002/mbo3.1369 37379428 PMC10291228

[26] Xu Q, Vandenkoornhuyse P, Li L, Guo J, Zhu C, Guo S, Ling N, Shen Q. 2022. Microbial generalists and specialists differently contribute to the community diversity in farmland soils. J Adv Res 40:17–27. doi:10.1016/j.jare.2021.12.003 36100325 PMC9481938

[27] von Meijenfeldt FAB, Hogeweg P, Dutilh BE. 2023. A social niche breadth score reveals niche range strategies of generalists and specialists. Nat Ecol Evol 7:768–781. doi:10.1038/s41559-023-02027-7 37012375 PMC10172124

[28] Rodriguez V, Moskwa L-M, Oses R, Kühn P, Riveras-Muñoz N, Seguel O, Scholten T, Wagner D. 2022. Impact of climate and slope aspects on the composition of soil bacterial communities involved in pedogenetic processes along the Chilean Coastal Cordillera. Microorganisms 10:847. doi:10.3390/microorganisms10050847 35630293 PMC9143490

[29] Levy-Booth DJ, Campbell RG, Gulden RH, Hart MM, Powell JR, Klironomos JN, Pauls KP, Swanton CJ, Trevors JT, Dunfield KE. 2007. Cycling of extracellular DNA in the soil environment. Soil Biol Biochem 39:2977–2991. doi:10.1016/j.soilbio.2007.06.020

[30] Torti A, Lever MA, Jørgensen BB. 2015. Origin, dynamics, and implications of extracellular DNA pools in marine sediments. Mar Genomics 24:185–196. doi:10.1016/j.margen.2015.08.007 26452301

[31] Davey HM, Hexley P. 2011. Red but not dead? Membranes of stressed Saccharomyces cerevisiae are permeable to propidium iodide. Environ Microbiol 13:163–171. doi:10.1111/j.1462-2920.2010.02317.x 21199254

[32] Hammes F, Berney M, Egli T. 2011. Cultivation-independent assessment of bacterial viability. Adv Biochem Eng Biotechnol 124:123–150. doi:10.1007/10_2010_95 21069588

[33] Kuske CR, Banton KL, Adorada DL, Stark PC, Hill KK, Jackson PJ. 1998. Small-scale DNA sample preparation method for field PCR detection of microbial cells and spores in soil. Appl Environ Microbiol 64:2463–2472. doi:10.1128/AEM.64.7.2463-2472.1998 9647816 PMC106412

[34] Morales-García AL, Walton R, Blakeman JT, Banwart SA, Harding JH, Geoghegan M, Freeman CL, Rolfe SA. 2021. The role of extracellular DNA in microbial attachment to oxidized silicon surfaces in the presence of Ca^2+^ and Na. Langmuir 37:9838–9850. doi:10.1021/acs.langmuir.1c01410 34347486 PMC8397393

[35] Pietramellara G, Ascher J, Borgogni F, Ceccherini MT, Guerri G, Nannipieri P. 2009. Extracellular DNA in soil and sediment: fate and ecological relevance. Biol Fertil Soils 45:219–235. doi:10.1007/s00374-008-0345-8

[36] Nagler M, Podmirseg SM, Griffith GW, Insam H, Ascher-Jenull J. 2018. The use of extracellular DNA as a proxy for specific microbial activity. Appl Microbiol Biotechnol 102:2885–2898. doi:10.1007/s00253-018-8786-y 29423636 PMC5847193

[37] Ibáñez de Aldecoa AL, Zafra O, González-Pastor JE. 2017. Mechanisms and regulation of extracellular DNA release and its biological roles in microbial communities. Front Microbiol 8:1390. doi:10.3389/fmicb.2017.01390 28798731 PMC5527159

[38] Slon V, Hopfe C, Weiß CL, Mafessoni F, de la Rasilla M, Lalueza-Fox C, Rosas A, Soressi M, Knul MV, Miller R, et al.. 2017. Neandertal and denisovan DNA from pleistocene sediments. Science 356:605–608. doi:10.1126/science.aam9695 28450384

[39] Pedersen MW, De Sanctis B, Saremi NF, Sikora M, Puckett EE, Gu Z, Moon KL, Kapp JD, Vinner L, Vardanyan Z, Ardelean CF, Arroyo-Cabrales J, Cahill JA, Heintzman PD, Zazula G, MacPhee RDE, Shapiro B, Durbin R, Willerslev E. 2021. Environmental genomics of late pleistocene black bears and giant short-faced bears. Curr Biol 31:2728–2736. doi:10.1016/j.cub.2021.04.027 33878301 PMC7617452

[40] Ogram A, Sayler GS, Barkay T. 1987. The extraction and purification of microbial DNA from sediments. J Microbiol Methods 7:57–66. doi:10.1016/0167-7012(87)90025-X

[41] Paget E, Monrozier LJ, Simonet P. 1992. Adsorption of DNA on clay minerals: protection against DNaseI and influence on gene transfer. FEMS Microbiol Lett 97:31–39. doi:10.1111/j.1574-6968.1992.tb05435.x

[42] Thomsen PF, Willerslev E. 2015. Environmental DNA – an emerging tool in conservation for monitoring past and present biodiversity. Biol Conserv 183:4–18. doi:10.1016/j.biocon.2014.11.019

[43] Logemann J, Graue J, Köster J, Engelen B, Rullkötter J, Cypionka H. 2011. A laboratory experiment of intact polar lipid degradation in sandy sediments. Biogeosciences 8:2547–2560. doi:10.5194/bg-8-2547-2011

[44] White DC, Stair JO, Ringelberg DB. 1996. Quantitative comparisons ofin situ microbial biodiversity by signature biomarker analysis. J Ind Microbiol Biotechnol 17:185–196. doi:10.1007/BF01574692

[45] de Souza Sant’Ana A, Peña WEL, Alvarenga VO, Oteiza JM. 2014. Alicyclobacillus, p 42–53. In Batt CA, Tortorello ML (ed), Encyclopedia of food microbiology. Oxford: Academic Press.

[46] Feiner G. 2006. The microbiology of specific bacteria, p 595–615. In Feiner G (ed), Meat Products Handbook. Woodhead Publishing.

[47] Cortesão M, Fuchs FM, Commichau FM, Eichenberger P, Schuerger AC, Nicholson WL, Setlow P, Moeller R. 2019. Bacillus subtilis spore resistance to simulated mars surface conditions. Front Microbiol 10:333. doi:10.3389/fmicb.2019.00333 30863384 PMC6399134

[48] Lacap DC, Warren-Rhodes KA, McKay CP, Pointing SB. 2011. Cyanobacteria and chloroflexi-dominated hypolithic colonization of quartz at the hyper-arid core of the Atacama Desert, Chile. Extremophiles 15:31–38. doi:10.1007/s00792-010-0334-3 21069402 PMC3017302

[49] Kuhlman KR, Venkat P, La Duc MT, Kuhlman GM, McKay CP. 2008. Evidence of a microbial community associated with rock varnish at Yungay, Atacama Desert, Chile. J Geophys Res 113. doi:10.1029/2007JG000677

[50] Urzì C, Brusetti L, Salamone P, Sorlini C, Stackebrandt E, Daffonchio D. 2001. Biodiversity of Geodermatophilaceae isolated from altered stones and monuments in the Mediterranean basin. Environ Microbiol 3:471–479. doi:10.1046/j.1462-2920.2001.00217.x 11553237

[51] Normand P. 2006. Geodermatophilaceae fam. nov., a formal description. Int J Syst Evol Microbiol 56:2277–2278. doi:10.1099/ijs.0.64298-0 17012547

[52] Gambino M, Lepri G, Štovícek A, Ghazayarn L, Villa F, Gillor O, Cappitelli F. 2021. The tombstones at the monumental cemetery of Milano select for a specialized microbial community. Int Biodeterior Biodegradation 164:105298. doi:10.1016/j.ibiod.2021.105298

[53] Gu J-Y, Zang S-G, Sheng X-F, He L-Y, Huang Z, Wang Q. 2015. Burkholderia susongensis sp. nov., a mineral-weathering bacterium isolated from weathered rock surface. Int J Syst Evol Microbiol 65:1031–1037. doi:10.1099/ijs.0.000059 25575828

[54] Rosado T, Dias L, Lança M, Nogueira C, Santos R, Martins MR, Candeias A, Mirão J, Caldeira AT. 2020. Assessment of microbiota present on a Portuguese historical stone convent using high-throughput sequencing approaches. Microbiologyopen 9:1067–1084. doi:10.1002/mbo3.1030 32352657 PMC7294311

[55] Purahong W, Hossen S, Nawaz A, Sadubsarn D, Tanunchai B, Dommert S, Noll M, Ampornpan L-A, Werukamkul P, Wubet T. 2021. Life on the rocks: first insights into the microbiota of the threatened aquatic rheophyte Hanseniella heterophylla. Front Plant Sci 12:634960. doi:10.3389/fpls.2021.634960 34194446 PMC8238419

[56] Warren-Rhodes KA, Lee KC, Archer SDJ, Cabrol N, Ng-Boyle L, Wettergreen D, Zacny K, Pointing SB, NASA Life in the Atacama Project Team. 2019. Corrigendum: subsurface microbial habitats in an extreme desert mars-analog environment. Front Microbiol 10:69. doi:10.3389/fmicb.2019.02129 30873126 PMC6403490

[57] Mergelov N, Mueller CW, Prater I, Shorkunov I, Dolgikh A, Zazovskaya E, Shishkov V, Krupskaya V, Abrosimov K, Cherkinsky A, Goryachkin S. 2018. Alteration of rocks by endolithic organisms is one of the pathways for the beginning of soils on earth. Sci Rep 8:3367. doi:10.1038/s41598-018-21682-6 29463846 PMC5820250

[58] Székely AJ, Langenheder S. 2014. The importance of species sorting differs between habitat generalists and specialists in bacterial communities. FEMS Microbiol Ecol 87:102–112. doi:10.1111/1574-6941.12195 23991811

[59] Bell TH, Bell T. 2020. Many roads to bacterial generalism. FEMS Microbiol Ecol 97. doi:10.1093/femsec/fiaa240 33238305

[60] Deonalli D, Sharma R, Jangid K. 2017. Microbial community dynamics during soil ecosystem development. In Kalia VC, Shouche Y, Purohit HJ, Rahi P (ed), Mining of microbial wealth and MetaGenomics. Singapore: Springer Singapore.

[61] Sen A, Daubin V, Abrouk D, Gifford I, Berry AM, Normand P. 2014. Phylogeny of the class Actinobacteria revisited in the light of complete genomes. The orders “Frankiales” and micrococcales should be split into coherent entities: proposal of frankiales ord. Int J Syst Evol Microbiol 64:3821–3832. doi:10.1099/ijs.0.063966-0 25168610

[62] Idris H, Goodfellow M, Sanderson R, Asenjo JA, Bull AT. 2017. Actinobacterial rare biospheres and dark matter revealed in habitats of the Chilean Atacama Desert. Sci Rep 7:8373. doi:10.1038/s41598-017-08937-4 28827739 PMC5566421

[63] Bull AT, Idris H, Sanderson R, Asenjo J, Andrews B, Goodfellow M. 2018. High altitude, hyper-arid soils of the central-andes harbor mega-diverse communities of actinobacteria. Extremophiles 22:47–57. doi:10.1007/s00792-017-0976-5 29101684 PMC5770506

[64] Bay SK, Waite DW, Dong X, Gillor O, Chown SL, Hugenholtz P, Greening C. 2021. Chemosynthetic and photosynthetic bacteria contribute differentially to primary production across a steep desert aridity gradient. ISME J 15:3339–3356. doi:10.1038/s41396-021-01001-0 34035443 PMC8528921

[65] Compant S, Nowak J, Coenye T, Clément C, Ait Barka E. 2008. Diversity and occurrence of Burkholderia spp. in the natural environment. FEMS Microbiol Rev 32:607–626. doi:10.1111/j.1574-6976.2008.00113.x 18422616

[66] Coenye T, Vandamme P. 2003. Diversity and significance of Burkholderia species occupying diverse ecological niches. Environ Microbiol 5:719–729. doi:10.1046/j.1462-2920.2003.00471.x 12919407

[67] Peers MJL, Thornton DH, Murray DL. 2012. Reconsidering the specialist-generalist paradigm in niche breadth dynamics: resource gradient selection by Canada lynx and bobcat. PLoS One 7:e51488. doi:10.1371/journal.pone.0051488 23236508 PMC3517500

[68] Mariadassou M, Pichon S, Ebert D. 2015. Microbial ecosystems are dominated by specialist taxa. Ecol Lett 18:974–982. doi:10.1111/ele.12478 26251267

[69] Xu Q, Luo G, Guo J, Xiao Y, Zhang F, Guo S, Ling N, Shen Q. 2022. Microbial generalist or specialist: Intraspecific variation and dormancy potential matter. Mol Ecol 31:161–173. doi:10.1111/mec.16217 34626522

[70] Shange RS, Ankumah RO, Ibekwe AM, Zabawa R, Dowd SE. 2012. Distinct soil bacterial communities revealed under a diversely managed agroecosystem. PLoS One 7:e40338. doi:10.1371/journal.pone.0040338 22844402 PMC3402512

[71] Oren A. 2014. Halophilic archaea on earth and in space: growth and survival under extreme conditions. Phil Trans R Soc A 372:20140194. doi:10.1098/rsta.2014.0194 25368347

[72] Falb M, Pfeiffer F, Palm P, Rodewald K, Hickmann V, Tittor J, Oesterhelt D. 2005. Living with two extremes: conclusions from the genome sequence of Natronomonas pharaonis. Genome Res 15:1336–1343. doi:10.1101/gr.3952905 16169924 PMC1240075

[73] Jiang ZM, Deng Y, Han XF, Su J, Wang H, Yu LY, Zhang YQ. 2022. Geminicoccus flavidas sp. nov. and Geminicoccus harenae sp. nov., two IAA-producing novel rare bacterial species inhabiting desert biological soil crusts. Front Microbiol 13:1034816. doi:10.3389/fmicb.2022.1034816 36386637 PMC9659566

[74] Peng M, Jia HB, Wang QY. 2017. The effect of land use on bacterial communities in saline–alkali soil. Curr Microbiol 74:325–333. doi:10.1007/s00284-017-1195-0 28102441

[75] Liu M, Huang HQ, Bao SX, Tong YH. 2019. Microbial community structure of soils in Bamenwan mangrove wetland. Sci Rep 9:8406. doi:10.1038/s41598-019-44788-x 31182804 PMC6557889

[76] Khilyas IV, Sorokina AV, Elistratova AA, Markelova MI, Siniagina MN, Sharipova MR, Shcherbakova TA, D’Errico ME, Cohen MF. 2019. Microbial diversity and mineral composition of weathered serpentine rock of the Khalilovsky massif. PLoS One 14:e0225929. doi:10.1371/journal.pone.0225929 31830070 PMC6907791

[77] Chen RW, He YQ, Cui LQ, Li C, Shi SB, Long LJ, Tian XP. 2021. Diversity and distribution of uncultured and cultured gaiellales and Rubrobacterales in south China sea sediments. Front Microbiol 12:657072. doi:10.3389/fmicb.2021.657072 34220745 PMC8248818

[78] Meier LA, Krauze P, Prater I, Horn F, Schaefer C, Scholten T, Wagner D, Mueller CW, Kühn P. 2019. Pedogenic and microbial interrelation in initial soils under semiarid climate on James Ross Island, Antarctic Peninsula region. Biogeosciences 16:2481–2499. doi:10.5194/bg-16-2481-2019

[79] Qiu L, Zhang Q, Zhu H, Reich PB, Banerjee S, van der Heijden MGA, Sadowsky MJ, Ishii S, Jia X, Shao M, Liu B, Jiao H, Li H, Wei X. 2021. Erosion reduces soil microbial diversity, network complexity and multifunctionality. ISME J 15:2474–2489. doi:10.1038/s41396-021-00913-1 33712698 PMC8319411

[80] Demergasso C, Neilson JW, Tebes-Cayo C, Véliz R, Ayma D, Laubitz D, Barberán A, Chong-Díaz G, Maier RM. 2023. Hyperarid soil microbial community response to simulated rainfall. Front Microbiol 14:1202266. doi:10.3389/fmicb.2023.1202266 37779711 PMC10537920

[81] Alawi M, Schneider B, Kallmeyer J. 2014. A procedure for separate recovery of extra- and intracellular DNA from a single marine sediment sample. J Microbiol Methods 104:36–42. doi:10.1016/j.mimet.2014.06.009 24955890

[82] Martin M. 2011. Cutadapt removes adapter sequences from high-throughput sequencing reads. EMBnet j 17:10. doi:10.14806/ej.17.1.200

[83] Callahan BJ, McMurdie PJ, Rosen MJ, Han AW, Johnson AJA, Holmes SP. 2016. DADA2: high-resolution sample inference from Illumina amplicon data. Nat Methods 13:581–583. doi:10.1038/nmeth.3869 27214047 PMC4927377

[84] Quast C, Pruesse E, Yilmaz P, Gerken J, Schweer T, Yarza P, Peplies J, Glöckner FO. 2013. The SILVA ribosomal RNA gene database project: improved data processing and web-based tools. Nucleic Acids Res 41:D590–6. doi:10.1093/nar/gks1219 23193283 PMC3531112

[85] R Core Team: R. 2022. A language and environment for statistical computing. In . Vienna, Austria: R Foundation for Statistical Computing, Vienna, Austria.

[86] Wickham H. 2016. ggplot2: elegant graphics for data analysis. Springer-Verlag, New York.

[87] McMurdie PJ, Holmes S. 2013. Phyloseq: an R package for reproducible interactive analysis and graphics of microbiome census data. PLoS One 8:e61217. doi:10.1371/journal.pone.0061217 23630581 PMC3632530

[88] Dufrêne M, Legendre P. 1997. Species assemblages and indicator species:the need for a flexible asymmetrical approach. Ecol Monogr 67:345–366. doi:10.1890/0012-9615(1997)067[0345:SAAIST]2.0.CO;2

[89] Zink KG, Mangelsdorf K. 2004. Efficient and rapid method for extraction of intact phospholipids from sediments combined with molecular structure elucidation using LC?ESI-MS?MS analysis. Anal Bioanal Chem 380:798–812. doi:10.1007/s00216-004-2828-2 15480579

[90] Müller KD, Husmann H, Nalik HP. 1990. A new and rapid method for the assay of bacterial fatty acids using high resolution capillary gas chromatography and trimethylsulfonium hydroxide. Zent Bl Bakteriol 274:174–182. doi:10.1016/S0934-8840(11)80100-3 2128179

[91] Balkwill DL, Leach FR, Wilson JT, McNabb JF, White DC. 1988. Equivalence of microbial biomass measures based on membrane lipid and cell wall components, adenosine triphosphate, and direct counts in subsurface aquifer sediments. Microb Ecol 16:73–84. doi:10.1007/BF02097406 24201534

