# Supplementary material for: Inside the Atacama Desert: uncovering the living microbiome of an extreme environment
Source: Appl Environ Microbiol. 2024 Nov 14;90(12):e01443-24. doi: 10.1128/aem.01443-24 (PMC11653780; doi:10.1128/aem.01443-24)
Supplement: Supplemental material — Tables S1 to S9, Fig. S1 to S6, and supplemental methods. [file aem.01443-24-s0001.docx]

**Inside the Atacama Desert:** **Uncovering the living microbiome of an extreme environment**

***Supplement***

Alexander Bartholomäus^1†^, Steffi Genderjahn^1†^, Kai Mangelsdorf^2^, Beate Schneider^1,3#^, Pedro Zamorano^4^, Samuel P. Kounaves^5,6^, Dirk Schulze-Makuch^1,3,7^, Dirk Wagner^1,8*^

^1^GFZ German Research Centre for Geosciences, Section Geomicrobiology, 14473 Potsdam, Germany

^2^GFZ German Research Centre for Geosciences, Section Organic Geochemistry, 14473 Potsdam, Germany

^3^Center of Astronomy and Astrophysics, Technical University Berlin, 10623 Berlin, Germany

^4^Laboratorio de Microorganismos Extremófilos, University of Antofagasta, Antofagasta 02800, Chile

^5^Department of Chemistry, Tufts University, Medford, MA 02155, USA

^6^Department of Earth Science & Engineering, Imperial College London, London SW72AZ, United Kingdom

^7^Leibniz-Institute of Freshwater Ecology and Inland Fisheries (IGB), Department of Experimental Limnology, 16775 Stechlin, Germany

^8^University of Potsdam, Institute of Geosciences, 14476 Potsdam, Germany

^#^present affiliation: German Environment Agency, 12304 Berlin, Germany

^†^These authors contributed equally to this work.

*corresponding author: Dirk Wagner, email: dirk.wagner@gfz-potsdam.de, phone: +49 331 6264-28800

**Table S1:** Log10-transformed mean and standard error (SE) values of qPCR and PLFA analysis. N.d. means value was below the limit of detection. PLFA values with * showed measured values but are also below the detection limit and should be taken with care.

| **Site** | **eDNA** | **iDNA** | **eDNA SE** | **iDNA SE** | **PLFA** |
| --- | --- | --- | --- | --- | --- |
| **CS 0-5 cm** | 7.54 | 5.65 | 0.28 | 0.41 | 6.73 |
| **CS 20-30 cm** | 5.70 | 5.36 | 0.44 | 0.11 | 5.33 |
| **AL 0-5 cm** | n.d. | 3.16 | n.d. | 0.11 | 4.70 |
| **AL 20-30 cm** | n.d. | 3.82 | n.d. | 0.15 | 3.83* |
| **RS 0-5 cm** | n.d. | 3.49 | n.d. | 0.28 | 5.33 |
| **RS 20-30 cm** | 2.84 | 6.00 | 0.20 | 0.14 | 4.94 |
| **LB 0-5 cm** | n.d. | 5.12 | n.d. | 0.35 | 5.92 |
| **LB 20-30 cm** | n.d. | 4.82 | n.d. | 0.25 | 4.85 |
| **ME 0-5 cm** | 4.13 | 4.79 | 0.47 | 0.05 | 5.25 |
| **ME 20-30 cm** | n.d. | 3.87 | n.d. | 0.28 | 4.28* |
| **YU 0-5 cm** | n.d. | 4.01 | n.d. | 0.02 | 4.47* |
| **YU 20-30 cm** | 4.27 | 5.79 | 0.10 | 0.06 | 5.40 |

**Table S2:** Read statistics for the 72 samples, including the single steps of processing from input to final non-chimera reads using DADA2. DenoisedF and DenoisedR are reads statistics after denoising of forward and reverse reads.

| **Input** | **Filtered** | **DenoisedF** | **DenoisedR** | **Merged** | **Nonchim** | **SampleSite** | **Depth** | **DNAtype** | **Replicate** |
| --- | --- | --- | --- | --- | --- | --- | --- | --- | --- |
| 87921 | 66999 | 66423 | 66461 | 62083 | 57454 | AL | 20 | iDNA | 3 |
| 69070 | 56541 | 56148 | 56191 | 51914 | 44128 | AL | 20 | iDNA | 1 |
| 50021 | 37969 | 37580 | 37641 | 34685 | 29303 | AL | 0 | iDNA | 3 |
| 27236 | 22015 | 21810 | 21873 | 20137 | 17305 | AL | 20 | iDNA | 2 |
| 38555 | 31732 | 31451 | 31515 | 29194 | 27129 | AL | 0 | iDNA | 1 |
| 30484 | 25172 | 24783 | 24873 | 23062 | 20900 | AL | 0 | iDNA | 2 |
| 42827 | 34898 | 34692 | 34710 | 32623 | 29915 | AL | 20 | eDNA | 1 |
| 64921 | 52512 | 52154 | 52223 | 48618 | 45361 | AL | 20 | eDNA | 2 |
| 63265 | 51700 | 51330 | 51309 | 48457 | 44204 | AL | 0 | eDNA | 1 |
| 69295 | 55986 | 55405 | 55496 | 51636 | 44840 | AL | 0 | eDNA | 2 |
| 46144 | 38179 | 37853 | 37849 | 35428 | 32850 | AL | 20 | eDNA | 3 |
| 43142 | 31891 | 31643 | 31663 | 29635 | 26962 | AL | 0 | eDNA | 3 |
| 81087 | 63582 | 63078 | 63144 | 58954 | 53593 | CS | 20 | iDNA | 1 |
| 79942 | 62682 | 62160 | 62237 | 58168 | 54499 | CS | 20 | iDNA | 2 |
| 70193 | 55702 | 55169 | 55296 | 51099 | 46858 | CS | 20 | iDNA | 3 |
| 55692 | 44401 | 43917 | 43993 | 40058 | 37360 | CS | 0 | iDNA | 1 |
| 82435 | 65113 | 64233 | 64372 | 57860 | 53436 | CS | 0 | iDNA | 2 |
| 104469 | 80929 | 80213 | 80247 | 72404 | 67443 | CS | 0 | iDNA | 3 |
| 43987 | 34414 | 33962 | 34040 | 30424 | 28068 | CS | 0 | eDNA | 1 |
| 53216 | 40876 | 40161 | 40311 | 35106 | 32201 | CS | 0 | eDNA | 2 |
| 27967 | 21810 | 21526 | 21562 | 19221 | 17878 | CS | 0 | eDNA | 3 |
| 166134 | 126729 | 125863 | 126086 | 117704 | 106973 | CS | 20 | eDNA | 1 |
| 85877 | 67597 | 67040 | 67221 | 62807 | 57484 | CS | 20 | eDNA | 2 |
| 112777 | 86698 | 86021 | 86256 | 80305 | 72476 | CS | 20 | eDNA | 3 |
| 84877 | 64830 | 64535 | 64508 | 61169 | 56738 | LB | 20 | iDNA | 3 |
| 70339 | 57280 | 56925 | 56897 | 53241 | 49115 | LB | 20 | iDNA | 1 |
| 65058 | 52337 | 52077 | 52047 | 49048 | 46299 | LB | 20 | iDNA | 2 |
| 60663 | 47489 | 47197 | 47193 | 44345 | 39889 | LB | 0 | iDNA | 1 |
| 50058 | 39589 | 39412 | 39386 | 37485 | 34070 | LB | 0 | iDNA | 2 |
| 60818 | 49423 | 49152 | 49120 | 46063 | 42407 | LB | 0 | iDNA | 3 |
| 22915 | 18838 | 18641 | 18709 | 16862 | 14515 | LB | 20 | eDNA | 3 |
| 66310 | 53821 | 53201 | 53453 | 49070 | 43455 | LB | 0 | eDNA | 1 |
| 52818 | 43226 | 42945 | 42933 | 40365 | 37836 | LB | 0 | eDNA | 2 |
| 36381 | 30003 | 29789 | 29810 | 28048 | 26134 | LB | 0 | eDNA | 3 |
| 41660 | 34047 | 33795 | 33728 | 31751 | 29410 | LB | 20 | eDNA | 1 |
| 35647 | 29284 | 29034 | 29102 | 27259 | 25015 | LB | 20 | eDNA | 2 |
| 57425 | 46249 | 45818 | 45861 | 42507 | 40037 | ME | 0 | iDNA | 1 |
| 65365 | 52258 | 51791 | 51896 | 48212 | 44421 | ME | 0 | iDNA | 2 |
| 75982 | 59985 | 59397 | 59436 | 55212 | 52194 | ME | 0 | iDNA | 3 |
| 44595 | 36148 | 35886 | 35877 | 33732 | 31692 | ME | 20 | iDNA | 1 |
| 46566 | 37368 | 37054 | 37035 | 34095 | 31025 | ME | 20 | iDNA | 3 |
| 70200 | 56318 | 55927 | 55822 | 51495 | 46218 | ME | 20 | iDNA | 2 |
| 43676 | 34645 | 34292 | 34336 | 31962 | 30133 | ME | 0 | eDNA | 1 |
| 44811 | 36314 | 35974 | 35990 | 33461 | 31765 | ME | 0 | eDNA | 3 |
| 65220 | 51448 | 50906 | 51120 | 46183 | 40260 | ME | 0 | eDNA | 2 |
| 43004 | 35167 | 34884 | 34937 | 32213 | 28751 | ME | 20 | eDNA | 1 |
| 35409 | 29042 | 28771 | 28841 | 26589 | 23852 | ME | 20 | eDNA | 2 |
| 31321 | 25276 | 25115 | 25132 | 23411 | 21402 | ME | 20 | eDNA | 3 |
| 43232 | 33902 | 33685 | 33713 | 31633 | 29728 | RS | 20 | iDNA | 1 |
| 42816 | 33353 | 33129 | 33135 | 30824 | 28641 | RS | 20 | iDNA | 2 |
| 37286 | 27563 | 27344 | 27396 | 25359 | 23868 | RS | 20 | iDNA | 3 |
| 64997 | 50853 | 50398 | 50429 | 46960 | 42847 | RS | 0 | iDNA | 1 |
| 58741 | 46004 | 45798 | 45700 | 42973 | 39739 | RS | 0 | iDNA | 3 |
| 97213 | 74070 | 73681 | 73527 | 68474 | 61608 | RS | 0 | iDNA | 2 |
| 45661 | 35964 | 35720 | 35742 | 33507 | 31134 | RS | 0 | eDNA | 1 |
| 37951 | 30615 | 30336 | 30329 | 28507 | 27054 | RS | 0 | eDNA | 3 |
| 38362 | 31200 | 30956 | 30898 | 28961 | 27938 | RS | 0 | eDNA | 2 |
| 88766 | 72991 | 72523 | 72590 | 67385 | 61507 | RS | 20 | eDNA | 1 |
| 63200 | 51423 | 51013 | 51006 | 47280 | 42508 | RS | 20 | eDNA | 2 |
| 54728 | 44785 | 44370 | 44487 | 40529 | 31477 | RS | 20 | eDNA | 3 |
| 46787 | 35996 | 35785 | 35787 | 33252 | 31348 | YU | 20 | iDNA | 1 |
| 42056 | 30391 | 30217 | 30211 | 27634 | 25669 | YU | 20 | iDNA | 2 |
| 57785 | 44850 | 44627 | 44547 | 41350 | 38681 | YU | 20 | iDNA | 3 |
| 43107 | 33848 | 33619 | 33657 | 31169 | 28283 | YU | 0 | iDNA | 1 |
| 44785 | 34889 | 34673 | 34687 | 31892 | 29783 | YU | 0 | iDNA | 2 |
| 46517 | 36112 | 35923 | 35970 | 33763 | 31853 | YU | 0 | iDNA | 3 |
| 84447 | 66967 | 66438 | 66513 | 61237 | 56272 | YU | 20 | eDNA | 2 |
| 45608 | 37577 | 37291 | 37329 | 34898 | 32453 | YU | 20 | eDNA | 1 |
| 62345 | 50869 | 50512 | 50484 | 47095 | 42606 | YU | 20 | eDNA | 3 |
| 63686 | 53100 | 52559 | 52694 | 48832 | 44001 | YU | 50 | eDNA | 3 |
| 51731 | 42286 | 41903 | 42003 | 38977 | 35223 | YU | 0 | eDNA | 2 |
| 69050 | 57215 | 56846 | 56728 | 52622 | 48079 | YU | 0 | eDNA | 3 |
| 166134 | 126729 | 125863 | 126086 | 117704 | 106973 | **Max** |  |  |  |
| 58071 | 46120 | 45748 | 45787 | 42485 | 38846 | **Mean** |  |  |  |
| 22915 | 18838 | 18641 | 18709 | 16862 | 14515 | **Min** |  |  |  |
| 4239159 | 3366734 | 3339591 | 3342426 | 3101427 | 2835790 | **Sum** |  |  |  |


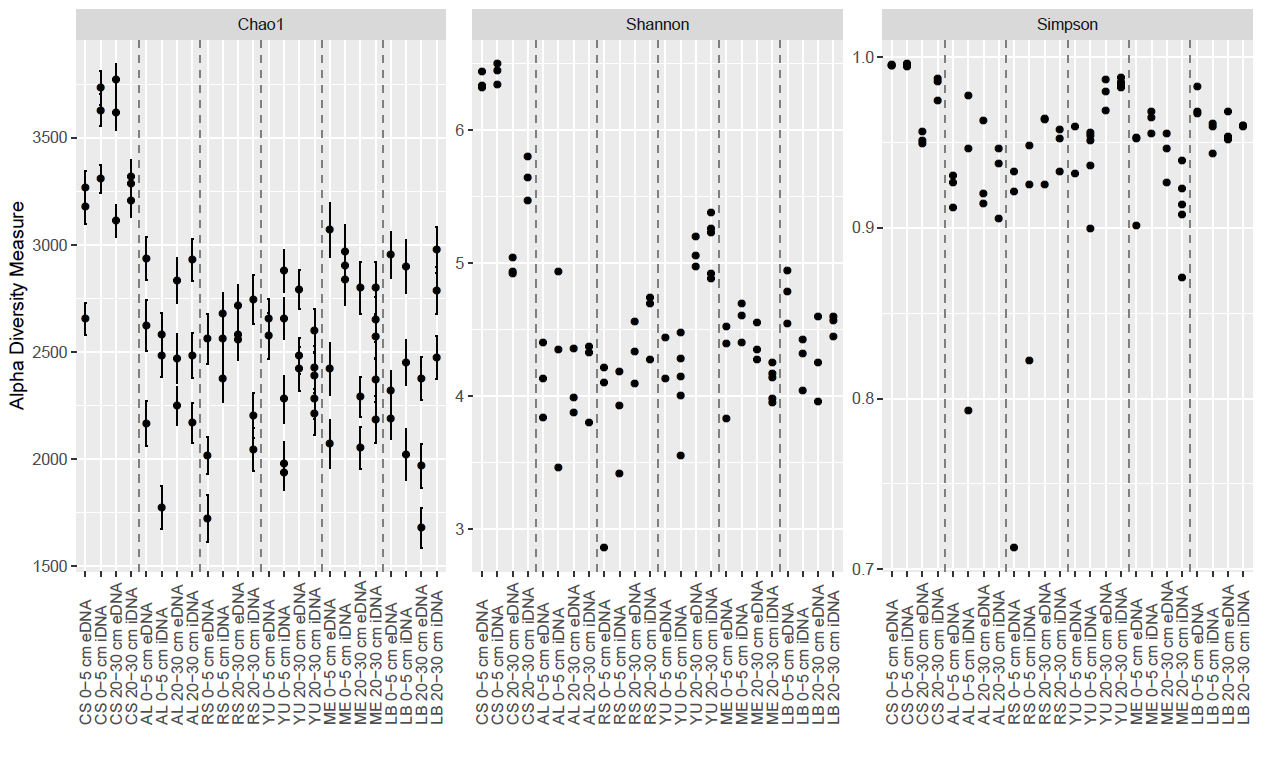
**Figure S1**: Alpha diversity measures Chao, Shannon’s H, and Simpsons index for all sites, depth and DNA types. Alpha diversity measures were calculated on a rarified ASV table without applying a relative abundance cutoff.


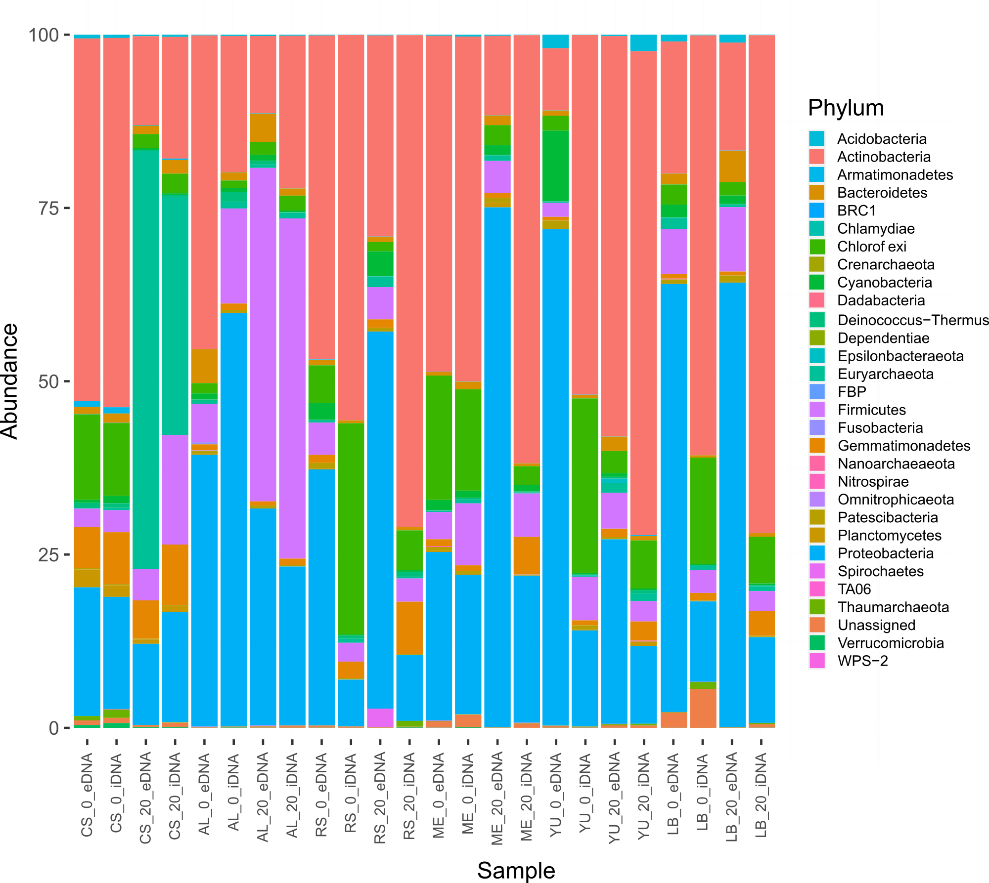


**Figure S2**: Diversity of the microbial community in the Atacama Desert showing the dominant phyla at two different depths. Study sites: Coastal Sand (CS), Aluvial Fan (AL), Red Sands (RS), Yungay (YU), Maria Elena (ME), and Lomas Bayas (LB).


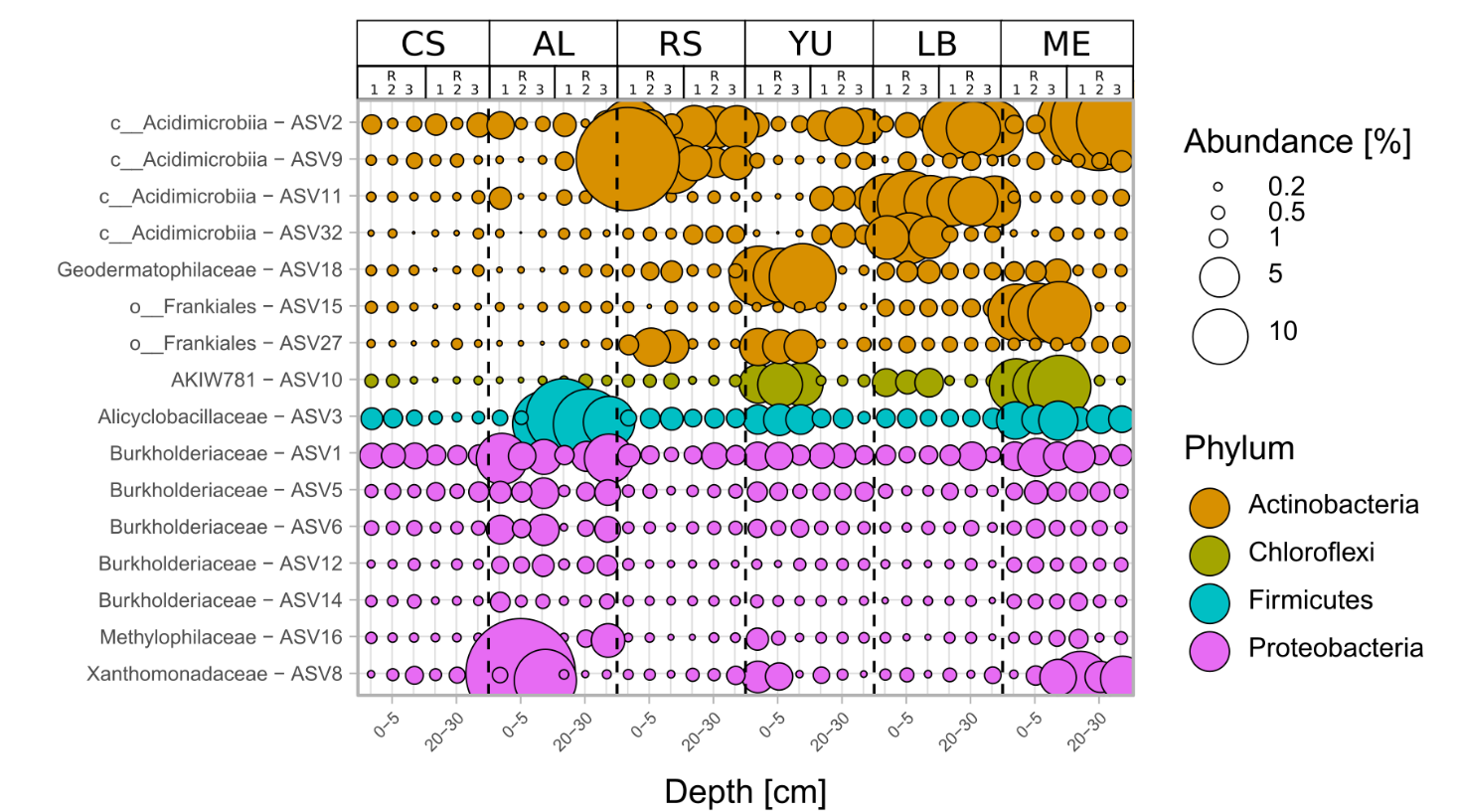


**Figure S3**: Bubble plot of iDNA bacterial generalists (≥ 90 % of sample site with relative abundance ≥ 0.1%) detected at the indicated depth intervals along the moisture transect: Coastal Sand (CS), Alluvial Fen (AL), Red Sands (RS), Yungay (YU), Maria Elena (ME), and Lomas Bayas (LB). Taxa are shown on the family or higher taxonomic level (c = class, o = order) with relation to each specific ASV, R = replicate.


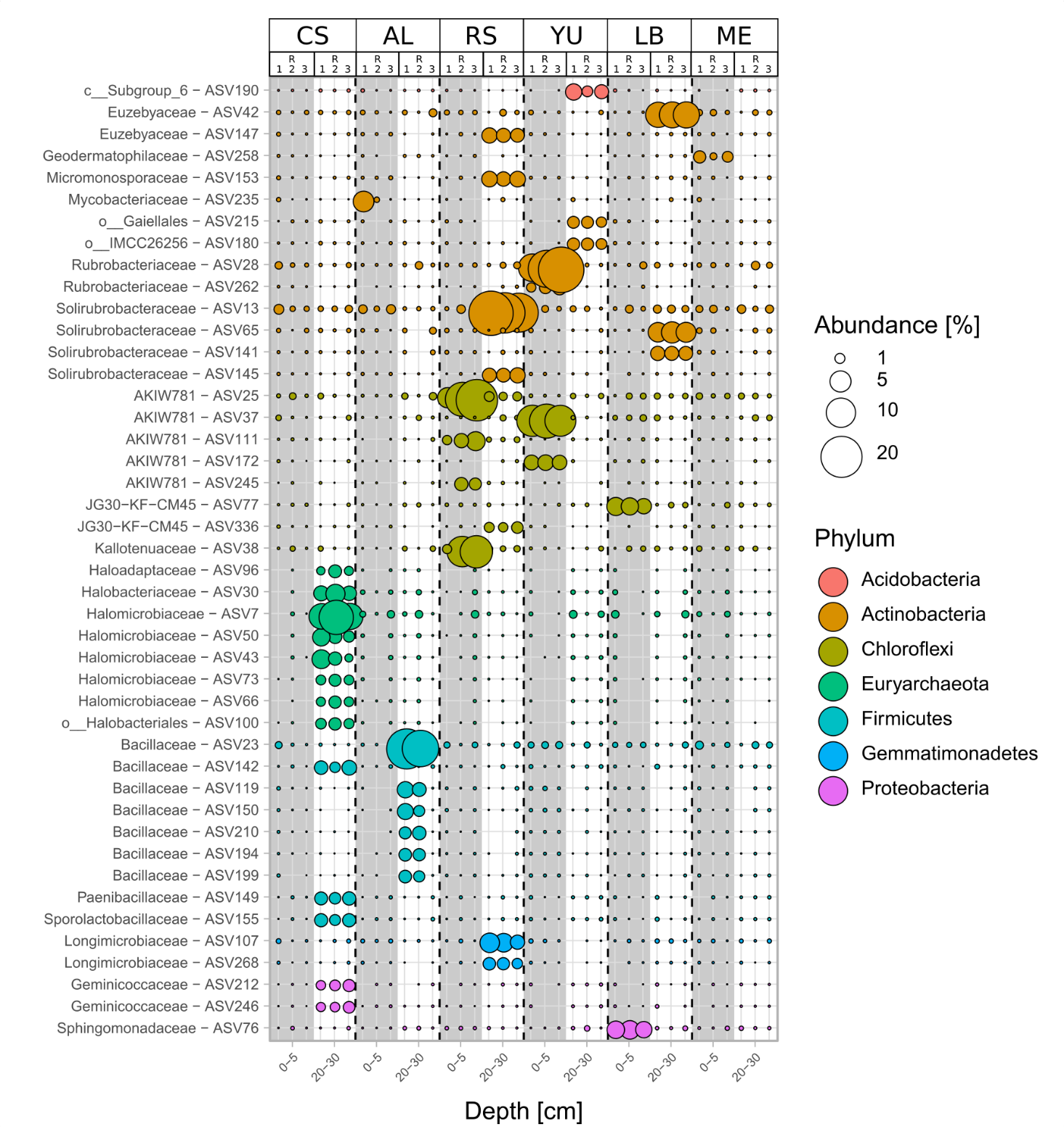


**Figure S4**: Bubble plot of iDNA bacterial specialists (indicator values > 0.8) detected at the indicated depth intervals along the moisture transect: Coastal Sand (CS), Alluvial Fen (AL), Red Sands (RS), Yungay (YU), Maria Elena (ME), and Lomas Bayas (LB). Taxa are shown on the family level or higher taxonomic level (c = class, o = order) with considering of each specific ASV, R = replicate.

| **DNA type** | **eDNA** | **iDNA** | **eDNA** | **iDNA** | **eDNA** | **iDNA** | **eDNA** | **iDNA** | **eDNA** | **iDNA** | **eDNA** | **iDNA** |
| --- | --- | --- | --- | --- | --- | --- | --- | --- | --- | --- | --- | --- |
| **Sample site** | **CS** | **CS** | **CS** | **CS** | **AL** | **AL** | **AL** | **AL** | **RS** | **RS** | **RS** | **RS** |
| **Depth** | **0** | **0** | **20** | **20** | **0** | **0** | **20** | **20** | **0** | **0** | **20** | **20** |
| Acidobacteria | 0.55 | 0.48 | 0.22 | 0.32 | 0.11 | 0.2 | 0.2 | 0.16 | 0.12 | 0.06 | 0.11 | 0.07 |
| Actinobacteria | 52.33 | 53.27 | 12.81 | 17.56 | 45.22 | 19.64 | 11.14 | 22 | 46.73 | 55.55 | 29.01 | 70.93 |
| Armatimonadetes | 0.83 | 0.9 | 0.09 | 0.21 | 0.02 | 0.03 | 0.05 | 0.03 | 0.07 | 0.02 | 0.03 | 0.04 |
| Bacteroidetes | 1.02 | 1.34 | 1.21 | 1.97 | 4.9 | 1.16 | 4.08 | 1.03 | 0.78 | 0.43 | 0.77 | 0.46 |
| BRC1 | 0.05 | 0.04 | 0 | 0 | 0 | 0 | 0 | 0 | 0 | 0 | 0 | 0 |
| Chlamydiae | 0 | 0 | 0 | 0 | 0 | 0.01 | 0 | 0 | 0.01 | 0 | 0 | 0 |
| Chloroflexi | 12.32 | 10.48 | 1.99 | 2.82 | 1.47 | 1.06 | 1.87 | 2.15 | 5.41 | 30.47 | 1.33 | 5.72 |
| Crenarchaeota | 0 | 0 | 0 | 0.01 | 0.02 | 0 | 0.01 | 0 | 0.01 | 0 | 0 | 0.01 |
| Cyanobacteria | 0.44 | 1.08 | 0.34 | 0.23 | 0.87 | 0.58 | 0.87 | 0.23 | 2.45 | 0.19 | 3.57 | 0.23 |
| Dadabacteria | 0 | 0 | 0 | 0 | 0 | 0 | 0 | 0 | 0 | 0 | 0 | 0 |
| Deinococcus-Thermus | 0.75 | 0.63 | 0.09 | 0.3 | 0.19 | 1.45 | 0.55 | 0.08 | 0.09 | 0.44 | 0.05 | 0.74 |
| Dependentiae | 0 | 0 | 0 | 0 | 0.01 | 0 | 0 | 0 | 0 | 0 | 0 | 0 |
| Epsilonbacteraeota | 0 | 0 | 0.01 | 0.01 | 0.05 | 0.02 | 0.01 | 0 | 0 | 0 | 0 | 0.03 |
| Euryarchaeota | 0.07 | 0.32 | 60.32 | 34.33 | 0.38 | 0.93 | 0.45 | 0.82 | 0.31 | 0.57 | 1.55 | 0.21 |
| FBP | 0.02 | 0.04 | 0 | 0 | 0 | 0 | 0 | 0 | 0 | 0 | 0 | 0 |
| Firmicutes | 2.6 | 3.18 | 4.51 | 15.8 | 5.56 | 13.71 | 48.11 | 49.08 | 4.65 | 2.72 | 4.65 | 3.34 |
| Fusobacteria | 0 | 0 | 0 | 0 | 0.3 | 0 | 0 | 0 | 0 | 0 | 0 | 0.01 |
| Gemmatimonadetes | 5.98 | 7.47 | 5.52 | 8.7 | 0.87 | 0.91 | 0.6 | 0.9 | 1.16 | 2.37 | 1.23 | 7.55 |
| Nanoarchaeaeota | 0 | 0 | 0 | 0.01 | 0 | 0 | 0 | 0 | 0 | 0 | 0 | 0 |
| Nitrospirae | 0 | 0.01 | 0 | 0.01 | 0 | 0 | 0 | 0 | 0 | 0 | 0 | 0 |
| Omnitrophicaeota | 0 | 0 | 0 | 0 | 0.07 | 0 | 0 | 0 | 0 | 0 | 0 | 0 |
| Patescibacteria | 0.18 | 0.17 | 0.13 | 0.11 | 0.44 | 0.31 | 0.34 | 0.14 | 0.75 | 0.17 | 0.47 | 0.09 |
| Planctomycetes | 2.57 | 1.67 | 0.6 | 0.93 | 0.13 | 0.1 | 0.08 | 0.1 | 0.19 | 0.06 | 0.06 | 0.06 |
| Proteobacteria | 18.57 | 16.18 | 11.71 | 15.85 | 39.21 | 59.67 | 31.32 | 22.93 | 36.93 | 6.74 | 54.39 | 9.49 |
| Spirochaetes | 0 | 0.07 | 0 | 0 | 0.03 | 0 | 0.06 | 0 | 0 | 0 | 2.64 | 0 |
| TA06 | 0 | 0 | 0 | 0 | 0 | 0 | 0 | 0 | 0 | 0 | 0 | 0 |
| Thaumarchaeota | 0.62 | 1.21 | 0.04 | 0.08 | 0.02 | 0.03 | 0.06 | 0.02 | 0.02 | 0.02 | 0.02 | 0.84 |
| Unassigned | 0.66 | 0.75 | 0.27 | 0.66 | 0.11 | 0.14 | 0.18 | 0.31 | 0.31 | 0.19 | 0.1 | 0.1 |
| Verrucomicrobia | 0.41 | 0.69 | 0.1 | 0.09 | 0.01 | 0.05 | 0.02 | 0.01 | 0.02 | 0.01 | 0.01 | 0.06 |
| WPS-2 | 0 | 0 | 0 | 0 | 0 | 0 | 0 | 0 | 0 | 0 | 0 | 0 |

**Table S3**: Phylum relative abundances

| **DNA type** | **eDNA** | **iDNA** | **eDNA** | **iDNA** | **eDNA** | **iDNA** | **eDNA** | **iDNA** | **eDNA** | **iDNA** | **eDNA** | **iDNA** |
| --- | --- | --- | --- | --- | --- | --- | --- | --- | --- | --- | --- | --- |
| **Sample site** | **ME** | **ME** | **ME** | **ME** | **YU** | **YU** | **YU** | **YU** | **LB** | **LB** | **LB** | **LB** |
| **Depth** | **0** | **0** | **20** | **20** | **0** | **0** | **20** | **20** | **0** | **0** | **20** | **20** |
| Acidobacteria | 0.16 | 0.27 | 0.17 | 0.08 | 1.96 | 0.06 | 0.21 | 2.38 | 0.98 | 0.11 | 1.14 | 0.08 |
| Actinobacteria | 48.46 | 49.74 | 11.43 | 61.75 | 8.96 | 51.87 | 57.75 | 69.82 | 19.04 | 60.55 | 15.57 | 71.81 |
| Armatimonadetes | 0.04 | 0.04 | 0.05 | 0.01 | 0.03 | 0.05 | 0.03 | 0.13 | 0.03 | 0.02 | 0.05 | 0.02 |
| Bacteroidetes | 0.52 | 1.1 | 1.4 | 0.39 | 0.74 | 0.49 | 2.07 | 0.62 | 1.55 | 0.34 | 4.49 | 0.51 |
| BRC1 | 0 | 0 | 0 | 0 | 0 | 0 | 0 | 0 | 0 | 0 | 0 | 0 |
| Chlamydiae | 0 | 0 | 0 | 0 | 0 | 0.01 | 0 | 0 | 0.02 | 0 | 0 | 0 |
| Chloroflexi | 17.92 | 14.61 | 2.9 | 2.73 | 2.17 | 25.18 | 3.17 | 6.94 | 2.89 | 15.38 | 1.95 | 6.72 |
| Crenarchaeota | 0 | 0 | 0.01 | 0 | 0 | 0.01 | 0.01 | 0 | 0 | 0 | 0 | 0.01 |
| Cyanobacteria | 1.5 | 1.05 | 1.4 | 0.91 | 10.17 | 0.23 | 0.54 | 0.22 | 1.83 | 0.09 | 1.23 | 0.28 |
| Dadabacteria | 0 | 0 | 0 | 0 | 0 | 0 | 0 | 0 | 0 | 0 | 0 | 0 |
| Deinococcus-Thermus | 0.06 | 0.28 | 0.07 | 0.15 | 0.04 | 0.07 | 0.27 | 0.44 | 0.08 | 0.14 | 0.06 | 0.11 |
| Dependentiae | 0 | 0 | 0 | 0 | 0 | 0.02 | 0 | 0 | 0 | 0 | 0 | 0 |
| Epsilonbacteraeota | 0.01 | 0 | 0.01 | 0.01 | 0 | 0.1 | 0.63 | 0 | 0.03 | 0 | 0 | 0.03 |
| Euryarchaeota | 0.2 | 0.51 | 0.77 | 0.13 | 0.2 | 0.12 | 1.42 | 1.12 | 1.62 | 0.57 | 0.38 | 0.7 |
| FBP | 0 | 0 | 0 | 0 | 0 | 0 | 0 | 0.02 | 0 | 0 | 0.01 | 0 |
| Firmicutes | 3.91 | 8.94 | 4.65 | 6.26 | 2 | 6.28 | 5.15 | 2.95 | 6.46 | 3.34 | 9.29 | 2.84 |
| Fusobacteria | 0.01 | 0 | 0 | 0 | 0.01 | 0 | 0.01 | 0 | 0 | 0 | 0 | 0 |
| Gemmatimonadetes | 0.98 | 0.8 | 0.7 | 5.41 | 0.53 | 0.72 | 1.22 | 2.76 | 0.65 | 1.11 | 0.57 | 3.6 |
| Nanoarchaeaeota | 0.14 | 0 | 0 | 0 | 0 | 0 | 0 | 0 | 0.13 | 0 | 0 | 0 |
| Nitrospirae | 0 | 0 | 0 | 0.02 | 0 | 0 | 0 | 0.14 | 0 | 0 | 0 | 0 |
| Omnitrophicaeota | 0 | 0 | 0 | 0 | 0 | 0 | 0 | 0 | 0 | 0 | 0 | 0 |
| Patescibacteria | 0.44 | 0.41 | 0.65 | 0.13 | 1.11 | 0.64 | 0.24 | 0.13 | 0.53 | 0.06 | 0.92 | 0.14 |
| Planctomycetes | 0.28 | 0.14 | 0.69 | 0.03 | 0.09 | 0.09 | 0.08 | 0.5 | 0.12 | 0.06 | 0.1 | 0.06 |
| Proteobacteria | 24.32 | 20.14 | 74.97 | 21.21 | 71.65 | 13.83 | 26.62 | 11.24 | 61.75 | 11.59 | 64.1 | 12.36 |
| Spirochaetes | 0 | 0 | 0 | 0.04 | 0 | 0.03 | 0.04 | 0 | 0 | 0.03 | 0 | 0 |
| TA06 | 0.05 | 0 | 0 | 0 | 0 | 0 | 0 | 0 | 0 | 0 | 0 | 0 |
| Thaumarchaeota | 0.06 | 0.05 | 0.02 | 0.08 | 0.02 | 0.05 | 0.18 | 0.26 | 0.06 | 1.01 | 0.05 | 0.2 |
| Unassigned | 0.9 | 1.83 | 0.08 | 0.62 | 0.29 | 0.13 | 0.35 | 0.33 | 2.21 | 5.61 | 0.06 | 0.5 |
| Verrucomicrobia | 0.02 | 0.07 | 0.02 | 0.01 | 0.01 | 0.02 | 0.01 | 0 | 0.01 | 0 | 0.01 | 0.02 |
| WPS-2 | 0.01 | 0 | 0 | 0 | 0 | 0 | 0 | 0 | 0 | 0 | 0 | 0 |

**Table S4**: Environmental parameters.

| **Name** | **CS_0** | **CS_20** | **AL_0** | **AL_20** | **RS_0** | **RS_20** | **YU_0** | **YU_20** | **LB_0** | **LB_20** | **ME_0** | **ME_20** |
| --- | --- | --- | --- | --- | --- | --- | --- | --- | --- | --- | --- | --- |
| **Sample site** | **CS** | **CS** | **AL** | **AL** | **RS** | **RS** | **YU** | **YU** | **LB** | **LB** | **ME** | **ME** |
| **Depth [cm]** | **0** | **20** | **0** | **20** | **0** | **20** | **0** | **20** | **0** | **20** | **0** | **20** |
| **Elevation [m]** | 506 | 506 | 854 | 854 | 1030 | 1030 | 1009 | 1009 | 1540 | 1540 | 1362 | 1362 |
| **Water content [% gravimetric at 60°C]** | 1.12 | 5.44 | 0.6 | 1.06 | 0.47 | 1.71 | 0.6 | 12.23 | NA | NA | NA | NA |
| **Water activity** | 0.9 | 0.85 | 0.6 | 0.9 | 0.5 | 0.9 | 0.9 | 0.9 | NA | NA | NA | NA |
| **pH** | 8.1 | 10.05 | 6.9 | 8.5 | 7.7 | 9.5 | 7.5 | 7.6 | 7.8 | 8.5 | 7.9 | 8.2 |
| **Electric conduct [S/m]** | 0.7 | 0.28 | 4.96 | 0.47 | 0.16 | 1.27 | 2.36 | 2.28 | 2.29 | 3.6 | 0.58 | 2.71 |
| **C [%]** | 0.053 | 0.081 | 0.016 | 0.007 | 0.018 | 0.037 | 0.061 | 0.040 | 0.246 | 0.18 | 0.035 | 0.050 |
| **N [%]** | 0.003 | 0.01 | 0.015 | 0.002 | 0.002 | 0.004 | 0.004 | 0.002 | 0.006 | 0.015 | 0.004 | 0.004 |
| **S [%]** | 0.028 | 0.011 | 0.144 | 0.022 | 0.06 | 0.064 | 0.64 | 3.9 | 0.55 | 1.03 | 0.058 | 3.5 |
| **Chloride [mmol/kg]** | 444 | 12440 | 10142 | 474 | 2 | 328 | 69 | 70 | 12 | 600 | 66 | 35 |
| **Sulfate [mmol/kg]** | 194 | 7317 | 3764 | 384 | 101 | 98 | 830 | 14980 | 12810 | 12575 | 2150 | 1169 |
| **Nitrate [mmol/kg]** | 45 | 850 | 468 | 28 | 6 | 34 | 88 | 69 | 63 | 246 | 85 | 44 |
| **Sodium [mmol/kg]** | 182 | 6310 | 6001 | 499 | 31 | 358 | 51 | 63 | 82 | 1046 | 55 | 267 |
| **Potassium [mmol/kg]** | 52 | 268 | 232 | 56 | 22 | 201 | 1437 | 0 | 98 | 361 | 50 | 263 |
| **Magnesium [mmol/kg]** | 25 | 551 | 290 | 0 | 3 | 4 | 47 | 62 | 39 | 47 | 21 | 58 |
| **Calcium [mmol/kg]** | 87 | 2259 | 1733 | 0 | 11 | 9 | 471 | 6040 | 5149 | 4712 | 699 | 5930 |
| **UV [J/m²]** | 2.15 | NA | 11.73 | NA | 12.73 | NA | 13.32 | NA | 15.35 | NA | 13.25 | NA |
| **UV final dose [J/m²]** | 52.40 | NA | 303.28 | NA | 289.01 | NA | 326.02 | NA | 355.71 | NA | 313.61 | NA |
| **relative humidity [%]** | 75.48 | NA | 27.3 | NA | 18.56 | NA | 8.0375 | NA | 9.65 | NA | 12.71 | NA |
| **Temp. rock [°C]** | 16.81 | NA | 25.78 | NA | 35.4 | NA | 37.44 | NA | 40.98 | NA | 37.99 | NA |
| **Temp. air [°C]** | 16.03 | NA | 28.76 | NA | 29.53 | NA | 34.76 | NA | 39.55 | NA | 35.93 | NA |

**Table S5**: Environmental parameters and ordination statistics, including axis direction, explained variance and P-value, calculated using the envfit function from vegan R package.

|  | **CA1** | **CA2** | **R^2^** | **P-value** |  |
| --- | --- | --- | --- | --- | --- |
| **UV** | 0.992 | 0.128 | 0.8494 | 0.001 | *** |
| **Relative Humidity** | -0.996 | -0.093 | 0.8285 | 0.001 | *** |
| **C** | 0.517 | 0.856 | 0.0257 | 0.659 |  |
| **N** | -0.975 | 0.224 | 0.0491 | 0.433 |  |
| **S** | 0.574 | 0.819 | 0.0921 | 0.186 |  |
| **pH** | -0.819 | 0.574 | 0.4253 | 0.001 | *** |
| **Electric conduct** | 0.969 | -0.248 | 0.0903 | 0.193 |  |
| **Elevation** | 0.964 | 0.267 | 0.5906 | 0.001 | *** |
| **Chloride** | -1.000 | -0.018 | 0.5028 | 0.001 | *** |
| **Sulfate** | -0.218 | 0.976 | 0.0908 | 0.2 |  |
| **Nitrate** | -0.999 | 0.038 | 0.5679 | 0.001 | *** |
| **Sodium** | -1.000 | -0.009 | 0.4226 | 0.002 | ** |
| **Potassium** | 0.255 | -0.967 | 0.3121 | 0.011 | * |
| **Magnesium** | -0.999 | 0.038 | 0.6189 | 0.001 | *** |
| **Calcium** | 0.232 | 0.973 | 0.1189 | 0.127 |  |
| **Temp. rock** | 0.966 | 0.259 | 0.7494 | 0.001 | *** |
| **Temp. air** | 0.998 | 0.070 | 0.7007 | 0.001 | *** |

**Table S6:** P-value for pairwise PERMANOVA to test community structure differences for all sample site with depth 0 cm and 20 cm combined. All sites differ significantly from each other.

|  | **AL** | **RS** | **ME** | **YU** | **LB** |
| --- | --- | --- | --- | --- | --- |
| **CS** | 0.001 | 0.001 | 0.001 | 0.001 | 0.001 |
| **AL** |  | 0.001 | 0.004 | 0.002 | 0.002 |
| **RS** |  |  | 0.001 | 0.001 | 0.001 |
| **ME** |  |  |  | 0.002 | 0.003 |
| **YU** |  |  |  |  | 0.002 |

**Table S7:** P-value for pairwise PERMANOVA to test community structure differences for eDNA and iDNA per site with depth 0 cm and 20 cm combined.

| **eDNA / iDNA** | **CS** | **AL** | **RS** | **ME** | **YU** | **LB** |
| --- | --- | --- | --- | --- | --- | --- |
| **CS** | 0.561 | 0.001 | 0.003 | 0.001 | 0.001 | 0.004 |
| **AL** | 0.003 | 0.28 | 0.001 | 0.01 | 0.014 | 0.002 |
| **RS** | 0.002 | 0.005 | 0.012 | 0.003 | 0.004 | 0.005 |
| **ME** | 0.002 | 0.002 | 0.001 | 0.032 | 0.004 | 0.001 |
| **YU** | 0.001 | 0.001 | 0.001 | 0.002 | 0.008 | 0.001 |
| **LB** | 0.005 | 0.006 | 0.004 | 0.008 | 0.009 | 0.004 |

**Table S8:** P-value for pairwise PERMANOVA to test community structure differences for different depth per site with eDNA and iDNA combined.

| **0-5cm / 20-30cm** | **CS** | **AL** | **RS** | **ME** | **YU** | **LB** |
| --- | --- | --- | --- | --- | --- | --- |
| **CS** | 0.003 | 0.002 | 0.006 | 0.001 | 0.001 | 0.002 |
| **AL** | 0.004 | 0.038 | 0.003 | 0.028 | 0.002 | 0.052 |
| **RS** | 0.005 | 0.004 | 0.002 | 0.004 | 0.001 | 0.008 |
| **ME** | 0.001 | 0.002 | 0.003 | 0.002 | 0.002 | 0.002 |
| **YU** | 0.003 | 0.001 | 0.002 | 0.012 | 0.001 | 0.014 |
| **LB** | 0.002 | 0.002 | 0.003 | 0.004 | 0.001 | 0.07 |


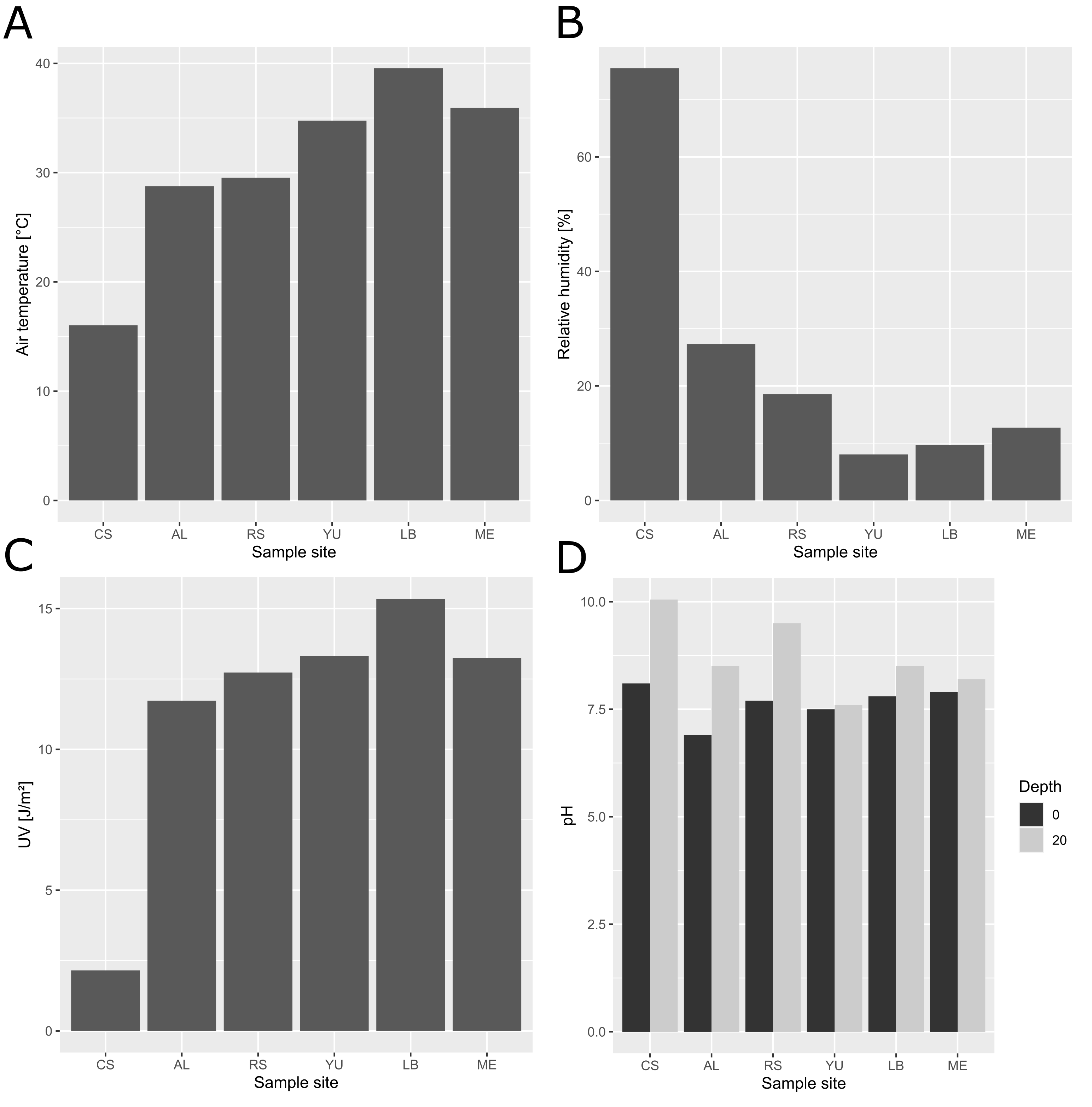


**Figure S5**: Selected environmental parameters air temperature, relative humidity, UV and pH. The full list of environmental parameters is available in Table S4 above. Study sites: Coastal Sand (CS), Aluvial Fan (AL), Red Sands (RS), Yungay (YU), Maria Elena (ME), and Lomas Bayas (LB).

Additional tests to show living and intact cells in iDNA fraction

*Methods*

**Live-dead staining**

iDNA fraction were obtained as described in the methods of the manuscript in triplicates by using around 3g soil from CS, RS and YU. For the staining the “Viability/Cytotoxicity Assay Kit for Bacteria Live & Dead Cells” (Biotium, CA, USA) was used. Shortly, staining mix (1 µl DMAO, 2 µl EthD-III) and 8 µl NaP buffer were mixed to obtain final staining mix. 1ml iDNA suspension was mixed with 1ml Nycodenz (50%) followed by 30 min by 1000g centrifugation. Supernatant was extracted, vortexed and centrifuged with 10.000g. Supernatant was removed and 100 µl NaP buffer added to pellet and 1 µl staining mix was added. 15 min incubation in the dark was carried out. 5 µl of final mix were used and given on microscopy slide.

**Microscopy**

Microscopy was done on a Zeiss AXIO microscope (Zeiss, Jena, Germany) and using the ZEN (blue edition, v3.14) software.

**Cultivation**

100 µl suspension of iDNA fraction was given to R2A plates and distributed equally. Incubation at 28°C for 6 days.

**16S rRNA PCR, SangerSequencing and BLAST**

To analyze the taxonomy of the colonies, DNA was extracted using DNeasy UltraClean Microbial Kit (QIAGEN Germany, Hilden, Germany) and a Polymerase Chain Reaction (PCR) targeting 16S rRNA using primer pair 27F (5’- gAgTTTgATCMTggCTCAg -3’; and 907R (5’- CCg TCA ATT CCT TTR AgT TT -3’) was performed. PCR was done using the following program in a Bio-Rad T100™ Thermal Cycler: a first denaturation step at 95°C for 5 min followed by 31 cycles at 95°C for 30 s, annealing at 56°C for 30 s, and extension at 72°C for 1 min. The PCR products were purified using the Agencourt AMPure XP kit (Beckman Coulter Life Science, Krefeld, Germany), pooled, and sent for sequencing at Eurofins Genomics (Ebersberg, Germany).

To obtain the best hit, BLAST was carried out using NCBI web services (<https://blast.ncbi.nlm.nih.gov/Blast.cgi>, accessed on 2024-05-27) using BLAST+ (v2.15.0) and nt BLAST against nr/nt database.

Results

We performed additional tests to show that the iDNA fraction includes living and intact cells. Both, live-dead straining (Fig. S6) and cultivation (Fig. S7) of the iDNA fraction support that the cells are indeed living. For each of the images (Fig. S6) from CS and RS only one dead cell is visible. The majority of the cells are living according to the staining. In addition, we plated the iDNA fraction (Fig. S7) to show single colonies that can grow. Finally, for some of the colonies a 16S rRNA PCR and sequencing was done. The best hits obtained from BLAST (Table S10) reveal that the colonies from genera that are also present in our ASV table.


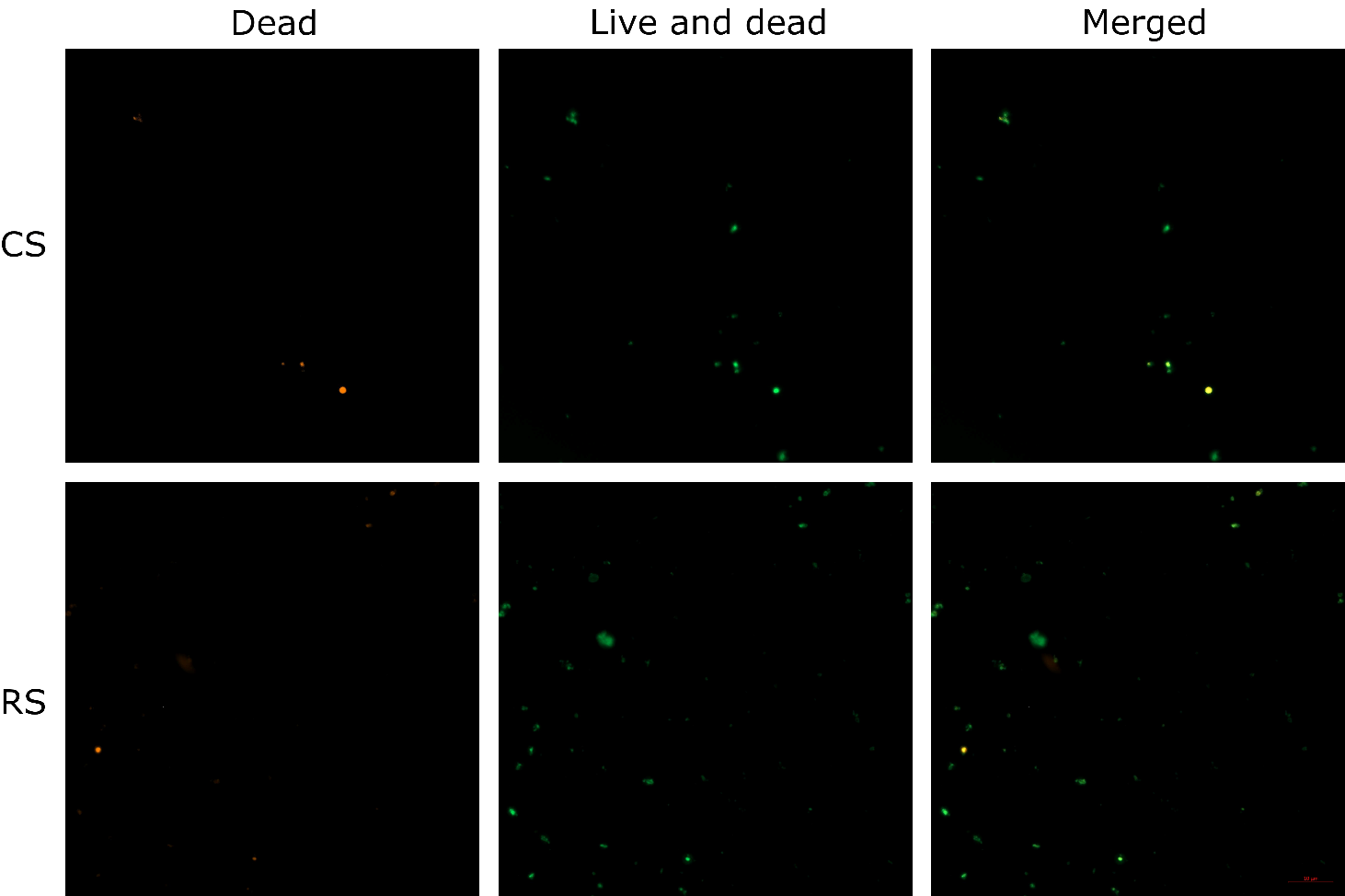


**Figure S6**: Live-dead straining of iDNA fraction for CS and RS. For CS one dead cell is visible in the low right. For RS one dead cell is visible at the left border of the image. All other cells look viable according to the staining.

|  | | |
| --- | --- | --- |
| **Sequence** | **Max identity %** | **Best hits** |
| CS-1 colony 1 | 99.4 | OQ225698.1 Pseudarthrobacter defluvii |
| CS-2 colony 1 | 98.82 | NR_025464.1 Kibdelosporangium aridum |
| CS-2 colony 2 | 99.22 | EU360598.1 Methylobacterium sp. XTB-8015 |
| CS-2 colony 3 | 96.09 | OQ300273.1 Pontibacter sp. strain R9_3 |
| RS-2 colony 1 | 97.18 | OP630767.1 Streptomyces sp. Strain |
| YU-2 colony 1 | 99.64 | KY753226.1 Streptomyces pulveraceus |

Table S9: BLAST results of the 16S rRNA sequencing from the plated colonies.
